# Foxd1-dependent induction of a temporal retinal character is required for visual function

**DOI:** 10.1242/dev.200938

**Published:** 2022-12-19

**Authors:** María Hernández-Bejarano, Gaia Gestri, Clinton Monfries, Lisa Tucker, Elena I. Dragomir, Isaac H. Bianco, Paola Bovolenta, Stephen W. Wilson, Florencia Cavodeassi

**Affiliations:** ^1^ Centro de Biología Molecular Severo Ochoa (CSIC-UAM), Madrid 28049, Spain; ^2^ Department of Cell and Developmental Biology, University College London, Gower Street, London WC1E 6BT, UK; ^3^ St George's University of London, Cranmer Terrace, London SW17 0RE, UK; ^4^ Department of Neuroscience, Physiology and Pharmacology, University College London, Gower Street, London WC1E 6BT, UK; ^5^ CIBER de Enfermedades Raras (CIBERER), Nicolás Cabrera 1, Madrid 28049, Spain

**Keywords:** *Foxd1*, *Rx3*, Retinal patterning, Fovea, *Area temporalis*, OKR, OMR, Zebrafish

## Abstract

Appropriate patterning of the retina during embryonic development is assumed to underlie the establishment of spatially localised specialisations that mediate the perception of specific visual features. For example, in zebrafish, an area involved in high acuity vision (HAA) is thought to be present in the ventro-temporal retina. Here, we show that the interplay of the transcription factor Rx3 with Fibroblast Growth Factor and Hedgehog signals initiates and restricts *foxd1* expression to the prospective temporal retina, initiating naso-temporal regionalisation of the retina. Abrogation of Foxd1 results in the loss of temporal and expansion of nasal retinal character, and consequent absence of the HAA. These structural defects correlate with severe visual defects, as assessed in optokinetic and optomotor response assays. In contrast, optokinetic responses are unaffected in the opposite condition, in which nasal retinal character is lost at the expense of expanded temporal character. Our study indicates that the establishment of temporal retinal character during early retinal development is required for the specification of the HAA, and suggests a prominent role of the temporal retina in controlling specific visual functions.

## Teaser

**Summary:** This study provides a mechanistic link between eye patterning and the establishment of functionally distinct retinal regions, and reveals that the temporal retina preferentially controls specific aspects of visual function.

## INTRODUCTION

The position at which retinal ganglion neurons differentiate along the naso-temporal and dorso-ventral axes of the forming eye determines the location at which they innervate central targets, ensuring accurate retinotopic connectivity. Consequently, the mechanisms by which retinal neurons acquire their regional identity are crucial for correct visual function. Regionally localised specialisations of the retina include the high acuity areas (HAAs) described in many diurnal organisms. In many primates and birds, this structure is called the fovea and is characterized by a morphological indentation, high density of RGCs and cone photoreceptors, absence of rod photoreceptors, specialised inner retina neuronal circuitry, and absence of vasculature (Bringmann et al., 2018; Bringmann, 2019; Da Silva and Cepko, 2017; Kolb et al., 2020; Kozulin et al., 2009, 2010). In teleost fish such as the zebrafish, some features associated with the fovea are evident in the ventro-temporal region of the retina, suggesting this region is comparable with the fovea in birds and primates (Mangrum et al., 2002; Pita et al., 2015; Schmitt and Dowling, 1999; Yoshimatsu et al., 2020; Zhou et al., 2020; Zimmermann et al., 2018). During development, the human fovea shows precocious expression of neuronal differentiation genes (Hoshino et al., 2017). In the chick, Da Silva and Cepko (2017) identified low levels of retinoic acid (RA) activity and high levels of the Fibroblast Growth Factor ligand FGF8 in a highly circumscribed area of the retina, prefiguring the fovea, a pattern that is conserved in the human presumptive fovea (Cornish et al., 2004, 2005; Da Silva and Cepko, 2017).

HAAs are generally located in the temporal retina (Bringmann, 2019; Kolb et al., 2020; Pita et al., 2015), suggesting their specification may be dependent upon acquisition of temporal character. In the zebrafish, the subdivision of the retina in domains with nasal and temporal character is evident from early stages of optic vesicle evagination by the expression of *foxg1* in the future nasal half and *foxd1* in the future temporal half of the eye (Hernández-Bejarano et al., 2015; Picker et al., 2009). Initially, the naso-temporal subdivision is aligned with the dorso-ventral axis of the developing central nervous system. However, as eye morphogenesis progresses, the eye primordium rotates such that nasal and temporal retinae relocate to their final position, aligned with the anterior-posterior axis (Kwan et al., 2012; Picker et al., 2009).

Previous studies have shown that the establishment of nasal and temporal character requires the spatially localised activity of the Sonic Hedgehog (Shh) and Fgf signalling pathways (Hernández-Bejarano et al., 2015; Picker and Brand, 2005; Picker et al., 2009). Shh, which is expressed in ventral midline structures, promotes *foxd1* expression in the ventral (future temporal) half of the optic primordium. Fgfs, which emanate from dorsal forebrain and adjacent tissues induce *foxg1* in the dorsal (future nasal) optic primordium and repress *foxd1* expression, contributing to its confinement ventrally. Cross-repression between Foxg1 and Foxd1 at the border between the dorsal and ventral halves of the eye primordium subsequently refines the naso-temporal subdivision (Hernández-Bejarano et al., 2015; Takahashi et al., 2009, 2003). When Shh signalling is absent, *foxd1* expression can be restored by suppressing Fgf signalling, indicating that an additional factor(s) can promote *foxd1* expression independently of Shh and Fgf (Hernández-Bejarano et al., 2015).

Here, we elucidate the molecular mechanisms involved in establishing temporal fate during retinal development and explore how alterations to naso-temporal retinal patterning impact HAA formation and visual function. We identify the transcription factor Rx3 (Loosli et al., 2003, 2001) as being required for *foxd1* expression in the forming eye. *rx3* is expressed in the eye field and is required for eye formation in all studied vertebrates (Andreazzoli et al., 1999, 2003; Bailey et al., 2004; Fish et al., 2014; Loosli et al., 2003, 2001; Mathers et al., 1997; Voronina et al., 2004). We show that the interplay of Rx3 with Shh and Fgf activities initiates and restricts *foxd1* expression to the prospective temporal half of the evaginating optic vesicles. We further show that the establishment of a *foxd1* expression domain in the eye is linked to the formation of the HAA. Larvae lacking Foxd1 function show normal retinal neuron differentiation and lamination, but temporal retinal and HAA markers are reduced/absent, and nasal markers expand throughout the retina. In contrast, fish in which nasal retinal character is absent and temporal character is expanded, show a concomitant expansion of HAA markers. In both conditions, retinotectal projections are severely perturbed. Despite this, only fish lacking Foxd1 and temporal retinal character show disrupted optokinetic and optomotor reflex behaviours that suggest impaired perception of whole-field visual motion. Our study reveals a prominent role of the temporal retina in controlling specific aspects of visual function and provides a mechanistic link between early patterning of the eye primordium and efficient visual performance.

## RESULTS

### Rx3 provides competence for Shh to promote *foxd1* expression in the ventral region of the optic vesicle

**Fig. 1.**
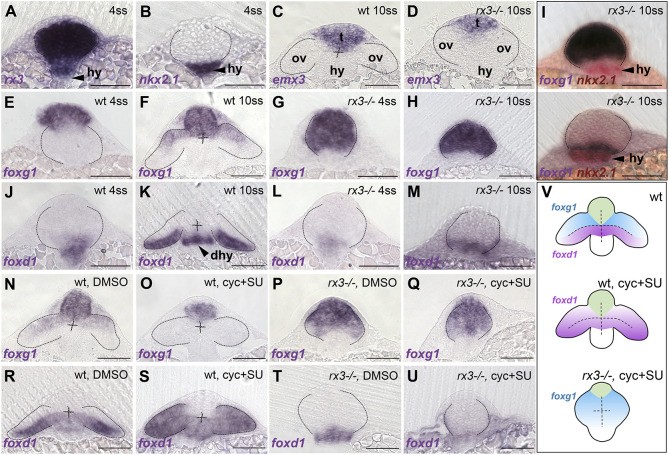
***foxd1* expression is reduced in *rx3^−/−^* mutant optic vesicles and lost in *rx3^−/−^* upon combined abrogation of Shh and Fgf.** (A-U) Frontal sections at the level of the forming telencephalon, optic vesicles and hypothalamus, with dorsal upwards; genotype, stage and/or treatment are indicated in the top right corner and the genes analysed are indicated in the bottom left corner. (A-D) Expression of *rx3* (A) and *nkx2.1* (B) in 4 ss wild-type/*rx3^−/−^* embryos (indistinguishable at this stage with a representative embryo shown) and *emx3* in 10 ss wild-type (C) and *rx3^−/−^* (D) embryos, highlighting the prospective telencephalic (t), hypothalamic (hy) and eye-forming (ov) domains. (E-H,J-M) Expression of *foxg1* (E-H) and *foxd1* (J-M) in 4 ss (E,G,J,L) and 10 ss (F,H,K,M) embryos. (I) Double *in situ* hybridisation of *foxd1* or *foxg1* and *nkx2.1* (red) to show the relationship between optic vesicles and the hypothalamus. (N-U) 10 ss stage embryos showing expression of *foxg1* (N-Q) or *foxd1* (R-U) in embryos treated with DMSO (N,P,R,T) or cyclopamine+SU5402 (O,Q,S,U). (V) Schematic representation of the conditions in R-U. Thirty to 50 embryos were treated and processed together per experiment and marker. *rx3^−/−^* embryos were recovered at the expected mendelian proportions and the phenotypes observed were fully penetrant. Scale bars: 100 µm. Dashed lines indicate the contour of the optic vesicles. dhy, dorsal hypothalamus; hy, hypothalamus; ov, optic vesicles; t, telencephalon.

The transcription factor Rx3 is specifically expressed in the eye field and is part of a network of regulatory proteins that confers eye fate and promotes the evagination of the optic vesicles. Lack of Rx function results in eye loss (Andreazzoli et al., 1999, 2003; Loosli et al., 2003; Mathers et al., 1997; Voronina et al., 2004). In zebrafish, the *rx3* mutant *chk^ne2611^* (*rx3^−/−^*; Loosli et al., 2003) bears a nonsense mutation in the homeodomain, leading to a stop codon in position 479 and a complete loss of Rx3 activity. Spatially localised expression of *rx3* (prospective retina marker), *nkx2.1* (prospective hypothalamic marker) and *emx3* (prospective telencephalic marker) is initially normal in the brain and eye-forming regions of *rx3^−/−^* embryos (Fig. 1A-D; Kennedy et al., 2004), indicating that, in the mutants, the eye-forming territory is still present and molecularly distinct from surrounding anterior neural structures. The presence of a prospective eye-forming domain has enabled us, in this study, to assess the effect of loss of *rx3* on expression of genes that delineate naso-temporal patterning.

*foxg1*, which is expressed in the telencephalon and nasal half of the eye primordium (Fig. 1E,F; Hernández-Bejarano et al., 2015), was expanded throughout most of the prospective eye domain in *rx3*^−/−^ mutants (Fig. 1G,H; Stigloher et al., 2006), whereas *foxd1* expression was much reduced (Fig. 1J-M). We confirmed the changes in the extent of *foxd1* and *foxg1* expression were localised to the prospective eye, by comparing their expression domains with those of *rx3* itself (which labels the eye field) and *nkx2.1* (Fig. 1A,B,I). A previous transcriptomic analysis in *rx3*^−/−^ mutants suggested that *foxd1* may be a downstream target of Rx3 (Yin et al., 2014). That study, together with our observations, support a scenario in which Rx3 is required for *foxd1* expression in the forming eye and provides cells with the competence to respond to Shh in promoting the expression of *foxd1*. If this is the case, the widespread expression of *foxd1* in the optic vesicle following loss of Shh and Fgf signalling should be absent in *rx3^−/−^* embryos. To address this, we first assessed whether Shh and Fgf signalling is overtly altered in *rx3^−/−^* mutants.

*rx3^−/−^* embryos showed normal levels of expression of genes encoding Fgf and Shh ligands, and target genes in the forebrain, suggesting that levels of activity of these two pathways in *rx3^−/−^* mutants are similar to wild types. The ligand encoding genes *fgf8* and *shh* showed only slight changes at the most anterior region of the forebrain [compare the extent of *fgf8* (brackets, Fig. S1A,B) and *shh* (arrows, Fig. S1G,H) expression between wild-type and *rx3^−/−^* embryos]. In addition, expression of *pea3* (*etv4*) and *erm* (*etv5b*) (direct targets of the Fgf pathway), and *gli1* and *patched 2* (direct targets of the Shh pathway), was largely normal (Fig. S1C-F,I-L). To abrogate Shh and Fgf activity in *rx3^−/−^* embryos, we treated clutches of embryos obtained from *rx3^+/−^* in-crosses simultaneously with two drugs: cyclopamine, which inhibits the Hh transducer Smoothened; and SU5042, an inhibitor of Fgf receptors (as previously described by Hernández-Bejarano et al., 2015).

In contrast to cyclopamine+SU5042-treated wild-type embryos in which *foxd1* expression is expanded (Fig. 1R,S; Hernández-Bejarano et al., 2015), the residual expression of *foxd1* in *rx3^−/−^* embryos (Fig. 1L,M,T) was completely lost when embryos were treated with cyclopamine+SU5042 (Fig. 1T-V; 30-50 embryos were treated and processed together per experiment; *rx3^−/−^* embryos were recovered at the expected mendelian proportions and the phenotypes observed were fully penetrant). Remarkably, *foxg1*, which was lost in the optic vesicles of wild-type embryos treated with cyclopamine+SU5402 (Fig. 1N,O), was expressed throughout the eye field of *rx3^−/−^* embryos regardless of treatment (Fig. 1P,Q,V). This may be due to the lack of the *foxg1* repressor Foxd1 in these conditions. Alternatively, it may be a consequence of the progressive adoption of telencephalic character by the eye-forming region of *rx3^−/−^* mutants (Stigloher et al., 2006). Consistent with the first scenario, *foxg1* was not expressed in the prospective eye region of *rx3^−/−^* embryos treated only with the Fgf inhibitor SU5402 (Fig. S2G-H), in which there was expanded *foxd1* expression (Fig. S2E,F).

### In the absence of *foxd1*, retinae fail to develop temporal character

**Fig. 2.**
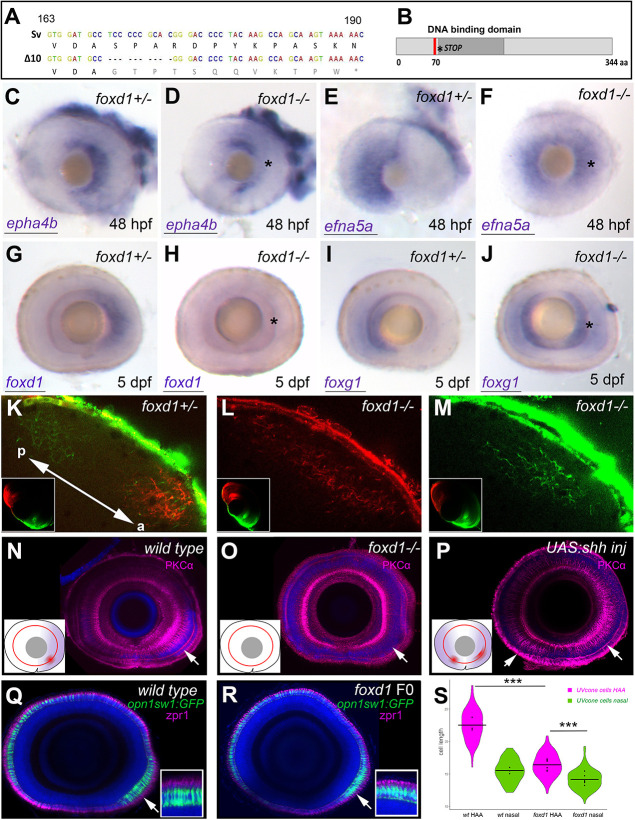
**Loss of *foxd1* results in retinae with expanded nasal and reduced temporal character.** (A) *foxd1* sequence comparison between nucleotides 163 and 190 of the open reading frame, highlighting the 10 bp deletion (Δ10, bottom row) in the *foxd1^cbm16^* mutant. (B) Diagrammatic representation of the Foxd1 protein, depicting (in red) the deleted region in the *cbm16* allele. The asterisks in A and B mark the position at which a newly generated stop codon is present. (C-J) Lateral views with nasal towards the left of 48 hpf (C-F) and 5 dpf (G-J) retinae, showing expression of nasal or temporal markers in *foxd1^−/−^* mutants and siblings. The markers assessed and the genotypes of the retinae are given in the bottom left and top right corners, respectively. Asterisks in D,F,H,J highlight altered expression in *foxd1* mutants. Twenty-five to 30 embryos were processed together per marker. *foxd1^−/−^* embryos were recovered at the expected mendelian proportions and the phenotypes observed were fully penetrant. Genotype of imaged embryos was confirmed by molecular genotyping. (K-M) Dorsal views of the tectum of *foxd1^−/−^* mutant (L,M; *n*=12) and sibling (K; *n*=10) embryos, with nasal RGC arbours labelled using DiO (green) and temporal RGC arbours using DiI (red). Insets in K-M show dorsal views of the corresponding eye. Tectum and eye orientation in K-M are indicated by the double-headed arrow in K (a, anterior; p, posterior). (N-P) PKCα immunostaining (magenta) in lateral views with nasal towards the left of 8 dpf wild-type (N), *foxd1^−/−^* mutant (O) and *tg{rx3:Gal4};UAS:Shh* (P) eyes. Nuclei are counterstained using DAPI (blue). Insets in N-P are schematics of the eyes in the corresponding panels, highlighting the extent of temporal character (purple), as assessed by gene expression, and the areas enriched with PKCα (red). Arrows indicate the position of PKCα enrichment. (Q,R) Sagittal sections across wild-type (Q) and *foxd1* crispant (R) eyes with nuclei stained using DAPI (blue), the outer photoreceptor segments stained using anti-Zpr1 antibodies (magenta) and UV cones expressing GFP [green, *Tg(opn1sw1:GFP)*] in 8 dpf larvae. (R) An example of a mild phenotype in comparison with other embryos with more severely reduced HAA labelling. (S) Violin plots showing differences in cone cell length between cells located in the HAA and nasal retina in wild type (*n*=3 eyes) and *foxd1* crispants (*n*=6 eyes). Horizontal bars show the mean cell length for each condition; dots indicate the mean cell length for each individual eye (***P<0.001; one-way ANOVA) Scale bars: 100 µm.

The results above indicate a crucial role for Rx3 and Shh in the induction of *foxd1* expression and the establishment of temporal character in the evaginating optic vesicle. Studies in other model organisms have shown that Foxd1 has a prominent role as determinant of retinal temporal character (Carreres et al., 2011; Hatini et al., 1994; Herrera et al., 2004; Takahashi et al., 2009, 2003). To further explore the role of *foxd1* in promoting temporal character, we generated a zebrafish loss-of-function mutant using CRISPR-Cas9 technology (Prykhozhij et al., 2017; see Materials and Methods). A 10 bp deletion immediately before the sequence encoding the Foxd1 DNA-binding domain led to a frame-shift and a stop codon in position 70 of the protein product (*foxd1^cbm16^*; Fig. 2A,B). This mutation likely leads to a complete loss of Foxd1 function.

*foxd1^−/−^* embryos developed at least up to 9 dpf, showing no apparent morphological malformations in the retina or elsewhere (data not shown). However, the temporal retinal marker *epha4b*, and *foxd1* itself, were downregulated in the mutant eyes (Fig. 2C,D,G,H). This downregulation was accompanied by a mirror-image duplication of expression of the nasal retinal markers *foxg1* and *efna5a* in the temporal half of the retina (Fig. 2E,F,I,J). The retinal phenotype of *foxd1^−/−^* mutants was phenocopied in F0 ‘crispants’ generated by simultaneous injection of three guides targeting different regions of the *foxd1* locus (Fig. S3M; Kroll et al., 2021; see Materials and Methods). All genotyped crispant embryos showed gene editing (*n*=16; Fig. S3N) and over 90% of the analysed embryos (65 out of 72) showed the expansion in *foxg1* expression that is characteristic of *foxd1* mutants (Fig. S3I,L).

The changes in the expression of naso-temporally restricted retinal markers in *foxd1^−/−^* mutants became gradually more evident as development progressed. Loss of *foxd1* expression was clear at late stages (Fig. 2G,H), but variable at early stages, with only a subset of mutant and crispant embryos at 12-14 hpf showing a moderate reduction in the *foxd1* domain (Fig. S3A-F). Ectopic expression of *foxg1* in the temporal retina of *foxd1^−/−^* mutants and crispants was only evident from 24 hpf (Fig. 2I,J, Fig. S3G-L and not shown). Before that stage, *foxg1* expression was normal in mutants and crispants (Fig. S3D-F). As there is no functional Foxd1 in the mutants/crispants, we believe that refinement of the *foxd1/foxg1* boundary by cross-repression does not occur and eventually *foxg1* expression expands into the temporal half of the eye. Of note, downregulation of *foxd1* expression in *foxd1^−/−^* embryos was not due to nonsense-mediated decay, as expression in other regions of the embryo was not affected (Fig. S4).

Analysis of retinotectal projections revealed an aberrant pattern of tectal innervation in *foxd1^−/−^* mutants. In control conditions, temporally located RGCs, injected with DiI, innervated the anterior region of the contralateral tectum, whereas nasally located RGCs, injected with DiO, innervated the posterior tectum (Fig. 2K; inset shows the corresponding eye, *n*=10 eyes). In *foxd1^−/−^* mutants, RGC terminals (labelled as for control conditions) from both nasal or temporal halves of the retina showed overlapping arborisations across much of the tectum (Fig. 2L,M; insets show the corresponding eye, *n*=12 eyes). This result indicates that, in the absence of RGCs with temporal character, axons from RGCs with nasal character expand their arbours throughout the tectum (Suetterlin et al., 2012). Overall, these results suggest that the *foxd1^−/−^* mutant retina has all-nasal character.

### HAA specification is linked to naso-temporal patterning

In fish, the ventro-temporal region of the retina (also called the area temporalis; Schmitt and Dowling, 1999) bears several structural specialisations that are thought to provide high visual acuity and support prey detection: a high density of U-cones (invested in detecting light in the ultraviolet spectrum) and low density of rods (Yoshimatsu et al., 2020; Zimmermann et al., 2018); a specialised inner retina neuronal circuit (Mangrum et al., 2002; Pita et al., 2015); and a characteristic number, shape and position of synaptic terminals at the inner plexiform layer (Zhou et al., 2020; Zimmermann et al., 2018). Immunostaining with anti-PKCα highlights the cell anisotropies found in the inner nuclear layer and inner plexiform layer at the level of the HAA (Zimmermann et al., 2018), while analysis of UV cone morphology reveals that those in the HAA are up to ten times longer than UV cones elsewhere in the retina (Yoshimatsu et al., 2020).

In contrast to wild-type zebrafish, where PKCα immunostaining was enriched in the ventro-temporal region of the retina (Fig. 2N, Movie 1), PKCα enrichment was reduced or lost in *foxd1^−/−^* retinae (Fig. 2O, Movie 2), suggesting that HAA-specific features are absent in *foxd1^−/−^* mutants. Analysis of UV cone morphology, visualised with *Tg(opn1sw1:GFP)*, further suggested a reduction of HAA-specific features in the absence of Foxd1 activity. Indeed, UV cone length in the HAA region of *foxd1* crispants was significantly shorter than in wild-type embryos and, even though still longer than the UV cones located in the nasal part of the retina, the difference was substantially reduced [Fig. 2Q-S; mean difference in length of 7.003 pixels between wild-type HAA and nasal cells (95% family-wise confidence interval: 6.15-7.855, *P*<0.001; *n* =78 cells each for wild-type HAA and nasal regions from *n*=3 eyes) and 2.250 pixels in *foxd1* crispants (95% confidence interval: 1.771-2.729, *P*<0.001, 156 cells each for *foxd1* crispant regions from *n*=6 eyes); one-way analysis of means, not assuming equal variances (*F*= 279.35, num d.f.=3.00, denom d.f.=200.85, *P*-value<2.2e-16), followed by Games-Howell post hoc testing].

Despite this reduction of HAA-specific features, *foxd1^−/−^* retinae showed an otherwise normal architecture (Fig. 2, Fig. S5; Movies 3-6). The distribution of R- and G-cones (as revealed by zpr1 immunostaining, Fig. S5A,B), amacrine and retinal ganglion cells (as revealed by ChAT and Islet 1 immunostaining, Fig. S5A-D) was largely normal and lamination was not affected. Optic nerve integrity and chiasma organisation were not overtly affected (Fig. S5E,F).

To determine whether changes in HAA markers are disrupted in other conditions that affect naso-temporal patterning, we analysed the opposite condition, in which embryos present a retina with all-temporal character. To generate this condition, we misexpressed Shh throughout the developing eye using the Gal4/UAS system (Distel et al., 2010; Halpern et al., 2008; Paquet et al., 2009), as previously described (Hernández-Bejarano et al., 2015). As shown in a previous report (Hernández-Bejarano et al., 2015), *Tg{rx3:Gal4};UAS:Shh* embryos showed an expansion of *foxd1* expression throughout the retina at the expense of *foxg1.*

*Tg{rx3:Gal4};UAS:Shh* retinae showed a dorso-temporal expansion in the HAA-associated enrichment of PKCα (Fig. 2P, arrows; compare with wild type in Fig. 2N), suggesting that the HAA was not only present, but expanded in this condition. Notably, an ectopic patch of PKCα enrichment also appeared to be present in the ventro-nasal half of the retina (Fig. 2P). Overall, these results suggest that the establishment of nasal and temporal character is linked to the formation of retinal specialisations such as the HAA.

### Visual function is impaired in foxd1 mutants

Perturbation of naso-temporal patterning and retinotectal projections in *foxd1^−/−^* mutants is likely to affect visual function. We therefore examined two robust visually guided stabilisation reflexes elicited by whole-field motion: the optokinetic and the optomotor responses (OKR and OMR, respectively). The OKR is a visually driven compensatory eye movement that minimises retinal image slip to stabilise gaze. During OKR, animals perform slow phase eye rotations in the direction of visual motion with intermittent fast-phase saccades to reset eye position. In the OMR, zebrafish larvae turn and swim in the direction of perceived whole field motion (Neuhauss et al., 1999).

**Fig. 3.**
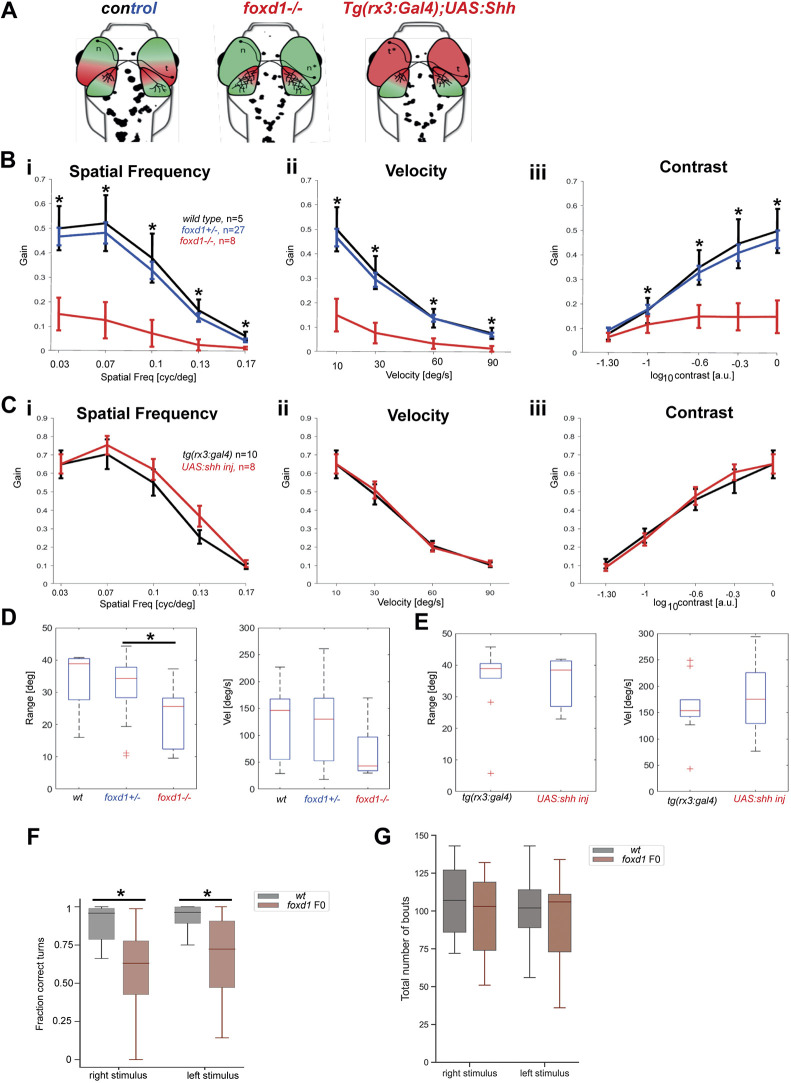
**Optokinetic response assays reveal severe visual deficiencies in *foxd1^−/−^* but not *tg{rx3:Gal4};UAS:Shh* embryos.** (A) Diagrammatic representation of the changes in naso-temporal pattern and retinotectal projections observed in the four categories of fish tested by optokinetic response (OKR). Genotype is provided at the top of each diagram. (B,C) Graphs detailing eye gain (expressed in a 0-1 range) in response to changes in spatial frequency (expressed in cycles per screen; Bi,Ci), grating velocity (expressed in degrees per second; Bii,Cii) and contrast (expressed in a 0-1 range; Biii,Ciii) in wild type (black, *n*=5 in B), *foxd1^+/−^* (blue, *n*=27 in B), *foxd1^−/−^* (red, *n*=8; in B), *tg{rx3:Gal4}* [black (rx3:gal4), *n*=10 in C] and *tg{rx3:Gal4};UAS:Shh* [red, (+UAS:shh), *n*=8 in C]. Graphical representation was generated using GraphPad Prism5 (data were analysed using two-way ANOVA; *P<0.001). (D,E) Range (expressed in degrees) of slow phase eye movement (left graphs) and peak velocity (expressed in degrees per second) during fast phase eye movement (right graphs) in *foxd1^−/−^* versus wild-type and *foxd1^+/−^* fish (D), and *tg{rx3:Gal4}* versus *tg{rx3:Gal4};UAS:Shh* fish (E) (*P<0.05; one-way ANOVA). (F) Fraction of correct turns for wild-type larvae (*n*=21) and *foxd1* crispants (*foxd1 F0*, *n*=21) when subjected to left- and right-oriented whole-field motion stimuli. Only directional bouts (i.e. left- and rightward swims, without forward swims) were considered for quantification. (G) Total number of bouts for wild-type larvae (*n*=21) and *foxd1* crispants (*foxd1 F0*, *n*=21) when subjected to left- and right-oriented whole-field motion stimuli.

*foxd1^−/−^* mutants were substantially impaired in OKR assays. We presented 6 dpf tethered larvae with rotating visual gratings and quantified the gain (ratio of eye velocity to stimulus velocity) of the slow phase of the optokinetic nystagmus as a function of stimulus spatial frequency, temporal frequency and contrast. Gain was reduced by around 80% in all conditions in *foxd1^−/−^* mutants (Fig. 3B, red) when compared with wild types and heterozygote siblings (Fig. 3B, black/blue; two-way ANOVA, main effect of genotype on spatial frequency *P*=2.3×10^−12^, temporal frequency *P*=4.1×10^−10^ and contrast *P*=8.8×10^−10^). Although this could reflect deficits in visual perception, we also observed a reduction in oculomotor range (Fig. 3D; one-way ANOVA, oculomotor range *P*=0.016, peak eye velocity *P*=0.16) suggesting that oculomotor defects might contribute to the phenotype. We therefore tested a second stabilisation reflex evoked by whole-field visual motion, and measured OMR performance. Whereas wild-type larvae robustly turned in the direction of whole-field motion, *foxd1* crispant larvae made significantly more mistakes (Fig. 3F; Kruskal–Wallis test; for both right and left stimulus, *P*<0.001). *foxd1* crispant larvae did not show evidence of locomotor dysfunction as they performed a similar total number of swim bouts compared with wild types (Fig. 3G; Kruskal–Wallis test; right stimulus, *P*=0.273; left stimulus, *P*=0.428) and displayed a normal range of turn angles (Fig. S6). Taken together, these data indicate that loss of Foxd1 function leads to impaired visual motion perception.

To determine whether impairment in visual motion perception is characteristic of other conditions that affect naso-temporal patterning and retinotectal projections, we assessed visual performance in *Tg{rx3:Gal4};UAS:Shh* fish with double temporal retinae. RGCs positioned in the nasal region of *Tg{rx3:Gal4};UAS:Shh* embryos projected more anteriorly in the tectum, overlapping the projections from temporally located RGCs (Fig. 3A; Hernández-Bejarano et al., 2015). Despite altered naso-temporal character and retinotectal projections, we did not observe any deficits in OKR performance in *Tg{rx3:Gal4};UAS:Shh* larvae (Fig. 3C, red; compare with uninjected *Tg{rx3:Gal4}* larvae in black; two-way ANOVA; main effect of genotype on spatial frequency, *P*=0.16; temporal frequency, *P*=0.85; contrast, *P*=0.85). These results suggest that the difference in OKR performance in *foxd1^−/−^* mutants and *Tg{rx3:Gal4};UAS:Shh* embryos is associated with the absence or presence, respectively, of temporal retinal character, and, potentially, with connectivity alterations.

## DISCUSSION

**Fig. 4.**
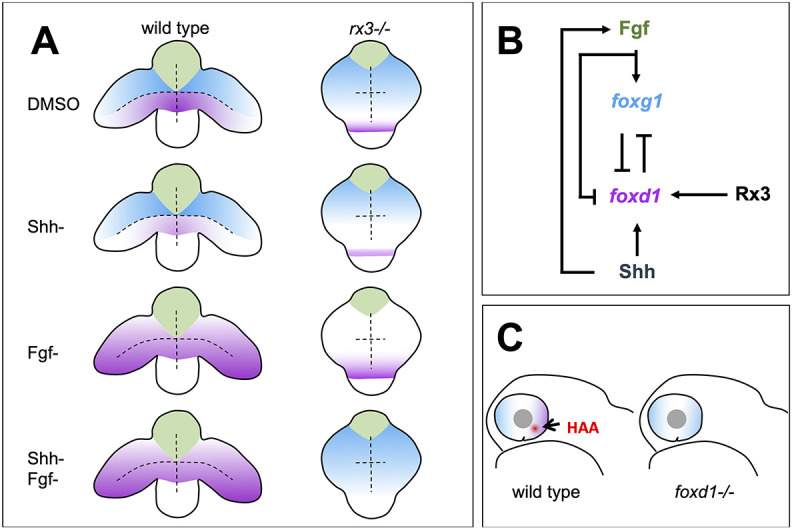
**Summary of roles for Rx3, Fgfs and Shh in establishment of nasal and temporal character in the developing eye primordium.** (A) Schematic representation of wild-type and *rx3^−/−^* embryos depicting the changes in *foxg1* (blue) and *foxd1* (magenta) expression upon abrogation of Shh and Fgf activities, singly or in combination. (B) Diagram detailing the proposed regulatory network controlling NT patterning. Shh promotes *foxd1* expression in the ventral (future temporal) half of the optic primordium. Fgfs induce *foxg1* in the dorsal (future nasal) optic primordium and repress *foxd1* expression, contributing to its confinement ventrally. Cross-repression between Foxg1 and Foxd1 at the border between the dorsal and ventral halves of the eye primordium subsequently refines the naso-temporal subdivision. Although induction of *shh* and *fgf8* expression occurs independently, *fgf8* expression is lower in the absence of Shh signalling (Hernández-Bejarano et al., 2015), suggesting Hh signalling initially promotes Fgf signalling. Rx3 induces *foxd1* expression and provides competence to optic vesicle cells to enhance *foxd1* expression in prospective temporal retina in response to Shh signalling. (C) Schematic representation of a differentiated optic cup with nasal (blue) and temporal (magenta) character, and the HAA (red) highlighted. The nasal character is expanded and HAA and temporal character are absent in *foxd1^−/−^* mutants (right) when compared with the wild type (left).

In this study we identify the eye-field specification gene *rx3* as a regulator of *foxd1* expression, thereby further resolving the transcriptional and signalling pathways that lead to naso-temporal retinal pattern (Fig. 4). We propose that *rx3* expression in the nascent eye field confers to this whole domain the competence to express *foxd1*. *foxd1* is repressed in the dorsal half of the eye primordium by Fgf activity and promoted in the ventral half by Hh activity (this study and Hernández-Bejarano et al., 2015). Fgf activity also promotes the expression of the nasal determinant *foxg1*, and Foxg1 and Foxd1 subsequently engage in a negative cross-regulatory relationship that refines and maintains the border between nasal and temporal retinal domains (Hernández-Bejarano et al., 2015).

The requirement of Rx3 to promote *foxd1* expression is most clearly revealed when the activity of other regulators is removed. In the absence of Fgf and Shh, *foxd1* expands throughout the optic vesicles. Removing Rx3 activity in this condition leads to loss of *foxd1* expression with a concomitant expansion of *foxg1* expression, confirming the requirement for Rx3 in the induction of *foxd1* expression. Our results do not allow us to determine whether Rx3 directly regulates *foxd1* expression. Even though previous studies identified potential Rx3-binding sites in the *foxd1* promoter, these regulatory sequences have not been validated (Yin et al., 2014). Loss of *rx3* function leads to a deregulation in the expression of other eye specification transcription factors and consequently the absence of *foxd1* expression in *rx3* mutants could either be a direct or an indirect consequence of the loss of Rx3 function.

An area temporalis in the zebrafish retina characterized by tightly packed cones was identified more than 20 years ago (Schmitt and Dowling, 1999). This area corresponds to the HAA, and more recent studies have shown that it bears a distinctive density of particular neuronal types and a specialised neuronal circuitry (Mangrum et al., 2002; Pita et al., 2015; Yoshimatsu et al., 2020; Zhou et al., 2020; Zimmermann et al., 2018). Our analysis suggests that the establishment of temporal retinal character is required for HAA formation. *foxd1^−/−^* mutants show an expansion of nasal retinal markers at the expense of temporal markers. Accumulation of PKCα in the ventro-temporal retina, which highlights the structural features of the HAA inner retina and inner plexiform layer (Zimmermann et al., 2018), and the distinctive morphology of UV cones in this region (Yoshimatsu et al., 2020) are compromised or lost in *foxd1^−/−^* mutants, suggesting that the mutant retina is devoid of at least some HAA features.

Not only the HAA, but also other structural anisotropies in the temporal retina may be affected in *foxd1^−/−^* mutants. Recent studies suggest that the OKR is driven mainly by stimuli covering the central visual field (Dehmelt et al., 2021) and predict that the HAA specialisations in the temporal retina may be dispensable for the OKR. However, OKR and OMR tests in *foxd1^−/−^* mutants reveal defective whole-field motion perception in these larvae. This suggests that either the HAA or other regions of temporal retina required for this visual response are affected in *foxd1^−/−^* mutants. Conversely, larvae with temporal character expanded into the nasal domain (*Tg{rx3:Gal4};UAS:Shh*) show an overtly normal OKR, suggesting a less prominent role of retina with nasal character in controlling at least some aspects of the OKR response.

Ablation and lesion experiments in both fish and other vertebrate models suggest that the execution of the OKR is driven mainly by the pretectum and can occur in the absence of an intact tectum (Flandrin and Jeannerod, 1981; Roeser and Baier, 2003). Here, we show that defective whole-field motion perception in *foxd1^−/−^* mutants, which show extensive disruption of retinotectal projections, is perturbed, but our analysis does not allow us to determine whether these two phenotypes are causally related as innervation of other retinorecipient areas may also be perturbed in *foxd1^−/−^* mutants. Indeed, some pretectal arborisation fields are preferentially innervated by the temporal retina (Robles et al., 2014), and our analysis did not determine whether these are affected in *foxd1^−/−^* mutants.

In summary, our study uncovers a mechanistic link between early naso-temporal patterning events in the eye primordium and the establishment of functionally distinct regions in the retina. Despite the importance of naso-temporal patterning for accurate RGC connectivity to specific retino-recipient areas, analysis of the visual function in *foxd1^−/−^* mutants and *Tg{rx3:Gal4};UAS:Shh* fish reveals a surprising robustness in the ability of the larvae to perceive stimuli in the absence of nasal or temporal-specific retinal character. These lines of fish provide an exciting new avenue to further our understanding of the role of nasal and temporal retinal character in mediating specific visual functions.

## MATERIALS AND METHODS

### Fish lines and husbandry

*AB* and *tupl* wild-type zebrafish strains, the transgenic lines *Tg{rx3::Gal4-VP16}^vu271Tg^* (Weiss et al., 2012), *Tg(opn1sw1:GFP)* (Takechi et al., 2003) and *Tg(atoh7:GFP)^rw021Tg^* (Masai et al., 2003)*, Tg(isl1:GFP)^rw0^* (Higashijima et al., 2000), and mutant the lines *chk^ne2611^* (Loosli et al., 2003) and *foxd1^cbm16^* were maintained and bred according to standard procedures (Aleström et al., 2020; Westerfield, 1993). All experiments conform to the guidelines from the European Community Directive and the British (Animal Scientific Procedures Act 1986) and to Spanish (Real Decreto 53/2013) legislation for the experimental use of animals.

### Generation of the *foxd1* mutant

The sequence to target in the *foxd1* open reading frame (ENSDARG00000029179) was selected using the UCSC Genome Browser (http://genome.ucsc.edu/). A DNA oligonucleotide bearing the target sequence (GCTTGTAGGGGTCCCGTGC; positions 435-417 on the reverse strand of the transcript), the T7 RNA polymerase-binding site and sequence complementary to the oligoB universal primer was commercially obtained, expanded with Expand High Fidelity DNA polymerase (Roche) and transcribed with the Maxi script T7 kit (NEB), following the manufacturers' instructions. Cas9 mRNA was generated by linearisation and transcription from the PCS2-nCas9n (Addgene, 47929) clone, and purified (PCR cleanup kit, Roche) and transcribed (SP6 mMessage mMachine Kit, Ambion) by following the manufacturers' instructions. F0 founders were generated by co-injection of guide RNA (25 ng/µl) and Cas9 mRNA (300 ng/µl) in *AB/tupl* embryos at the one-cell stage. Cleavage efficiency was assessed in pools of injected embryos by CRISPR-STAT analysis (Carrington et al., 2015). Genotyping of F0 founders and their progeny was performed by CRISPR-STAT or HRM analysis from genomic DNA samples obtained from tail fin biopsies. The primers used are detailed in Table S1.

### Generation of multi-guide *foxd1* crispants

The phenotype of the *foxd1^cbm16^* mutants was phenocopied by multi-guide injection, following a protocol previously published (Kroll et al., 2021). Three synthetic RNA guides were designed (1AA, 1AB and 1AC guides, Table S1) and ordered to Integrated DNA Technologies (IDT). Guides were annealed to the tracrRNA oligonucleotide (IDT#1072532), assembled with Cas9 protein (IDT#1081058) and injected into one-cell stage wild-type embryos, as previously described (Kroll et al., 2021). A subset of the injected embryos was genotyped by HRMA, using the HRMA primers described in Table S1, to confirm the presence of gene editing.

### Microinjection and drug treatments

Treatments with SU5402 and cyclopamine were performed following the protocols optimised and previously described by Hernández-Bejarano et al. (2015). SU5402 (Calbiochem) and cyclopamine (Calbiochem) were applied at a concentration of 10 µM and 100 µM, respectively, to pools of embryos derived from the mating of *rx3^+/−^* parental fish, at the corresponding stage of embryonic development. 30 to 50 embryos were treated and processed together per experiment/marker. *rx3^−/−^* embryos were recovered at the expected mendelian proportions and the phenotypes observed were fully penetrant.

Overexpression of *shh* in the optic vesicle under the control of the Gal4/UAS system (Halpern et al., 2008) was performed by injecting 20-30 ng of bidirectional GFP:UAS:*shh* plasmid DNA into the cell of one-cell stage *Tg{rx3::Gal4-VP16}^vu271Tg^* embryos. Only embryos with homogeneous GFP expression in the optic vesicles were selected and processed for analysis (as described by Hernández-Bejarano et al., 2015).

### mRNA detection and immunolabelling

mRNA detection (preparation of RNA antisense probes and whole-mount *in situ* hybridisation) was performed as previously described (Hernández-Bejarano et al., 2015). Immunolabelling of 8 dpf wild-type*, foxd1* and *Tg{rx3::Gal4};*UAS:*Shh* retinae using anti Zpr1(1:100, ZIRC, ZDB-ATB-081002-43), anti-GFP (Abcam, ab13970, 1:1000), anti-PKCα (Sigma, P4334, 1:100), Alexa-coupled secondary antibodies (Jackson ImmunoResearch, 1:500) and the nuclear dye DAPI was performed on RPE-dissected retinae as described previously (Zimmermann et al., 2018).

### Measurement of UV cones

UV cone cells were labelled with anti-GFP antibody in wild-type and *foxd1* crispant *tg(opn1sw1:GFP)* eyes. Individual cells located in five different *z*-planes covering 50 μm were manually measured using Fiji software (Schindelin et al., 2012). Selected HAA cells were located between 3 and 6 o'clock, whereas nasal cells were located between 6 and 9 o'clock. One-way analysis of means, not assuming equal variances, followed by Games-Howell post-hoc testing.

### Tracing of retinotectal projections

Nasal and temporal RGCs were labelled with DiO and DiI, respectively, in 6 dpf wild-type, *foxd1^−/−^* and *Tg{rx3:Gal4};*UAS:*Shh* retinae previously fixed in 4% paraformaldehyde. Labelled larvae were incubated at room temperature for at least 24 h before imaging. Each tectum and its corresponding eye were sequentially imaged (Hernández-Bejarano et al., 2015).

### Optokinetic response test

6 dpf larvae were anesthetised with tricaine (MS-222, Sigma), embedded in 1% low-melting point agarose (Sigma) and immersed in embryo medium. A small amount of agarose was removed from around the eyes to allow movement. Once larvae had recovered from anaesthesia, optokinetic response (OKR) performance was tested by presenting rotating visual gratings that varied in size, contrast and speed. We presented a set of 12 unique stimuli that was repeated twice for each animal. Horizontal eye movement was tracked under infrared illumination (850 nm) with an AVT Pike camera (100 frames per second). Stimulus presentation and machine vision were controlled using LabVIEW (National Instruments). Slow phase gain, saccadic eye velocity and oculomotor range were computed using custom MATLAB software (https://bitbucket.org/biancolab/okrsuite/src/master/). *foxd1^−/−^* mutants (*n*=8) were compared with wild type (*n*=5) and *foxd1^+/−^* heterozygotes (*n*=27) and *Tg{rx3:Gal4};*UAS:*Shh* (*n*=8) were compared with *Tg{rx3:Gal4}* (*n*=10) uninjected larvae. Statistical comparison of group means was performed using ANOVA with post-hoc pairwise tests corrected for multiple comparisons (Tukey-Kramer method; MATLAB, MathWorks).

### Optomotor response assay

6 dpf zebrafish larvae were individually placed in a 6 cm Petri dish and presented from below via a projector with moving grating stimuli in a closed-loop manner. The customized stimulus protocol and tracking of the freely moving fish was implemented using the Stytra software package (Štih et al., 2019). The time course of the assay consisted of five repetitions of rightwards and leftwards moving gratings (20 s each), separated by 10 s of static gratings (pause), for a total time of 5 min per fish. Twenty-one wild-type and 21 *foxd1*-crispant larvae were subjected to the optomotor response (OMR) assay. Custom behavioural analysis was implemented using Python and the Bouter package (Štih et al., 2022). A Kruskal–Wallis test was used for comparing group medians (SciPy package in Python).

### Imaging and data processing

DiI/DiO-traced embryos were embedded in low melting point agarose (Sigma) at 1-1.5% in PBS for confocal imaging using a 40× (0.8 NA) long-working distance water-immersion lens. A Zeiss LSM710 confocal microscopy system was used for image acquisition. Immunolabelled 8 dpf retinae were imaged using a Leica SP8 microscope with a 25× water-immersion lens.

*In situ* hybridised embryos and dissected eyes were mounted flat in a drop of glycerol and dorsal images were acquired with a 20× (0.70 NA) dry lens using a Leica CTR 5000 microscope connected to a digital camera (Leica DFC 500), and operated by Leica software. Some embryos were embedded in gelatine/BSA for vibratome sectioning, as previously described (Hernández-Bejarano et al., 2015; Sánchez-Arrones et al., 2013). Alternatively, embryos were cryoprotected in sucrose 30% and embedded in OCT (Sakura Fintek) for cryo-sectioning, as previously described (Cavodeassi et al., 2013). Sections (16 μm) were obtained using a Leica VT1000S vibratome or a Leica cryostat, mounted in glycerol and imaged with a 40× (1.3 NA) oil-immersion lens. Images in Fig. S3 were acquired using a 20× dry lens using a Nikon Eclipse microscope connected to a digital camera (DS-Fi3) and operated by Nikon software (NIS-Elements). Raw confocal images were processed with ImageJ. Processed images were exported as TIFF files and all figures were composed using Photoshop.

## Supplementary Material

10.1242/develop.200938_sup1 Supplementary information
