# Supplementary material for: Foxd1-dependent induction of a temporal retinal character is required for visual function
Source: Development. 2022 Dec 19;149(24):dev200938. doi: 10.1242/dev.200938 (PMC9845753; doi:10.1242/dev.200938)
Supplement: Supplementary information [file develop-149-200938-s1.pdf]

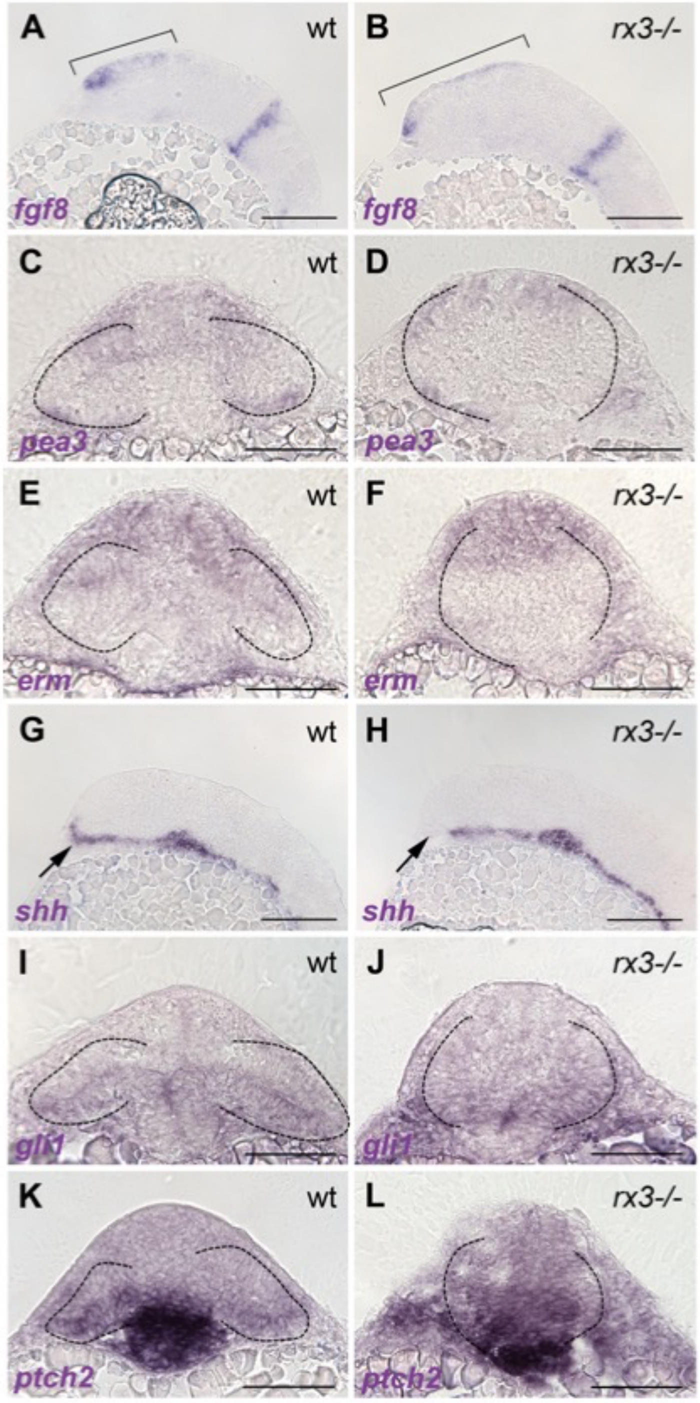

Fig. S1. expression of Fgf and Shh pathway genes is similar in *rx3*$^{-/-}$ mutants and siblings. (A-L) Sagittal (A,B; G,H) and frontal sections at the level of the forming telencephalon, optic vesicles and hypothalamus (C-F;I-L), with dorsal up. Genes analysed and genotypes are shown bottom left and top right respectively. Brackets in (A-B) highlight the extent of *fgf8* expression in the prospective telencephalon; arrows in (G-H) highlight the extent of *shh* expression in the anterior hypothalamus. All embryos are 10ss.

Development • Supplementary information

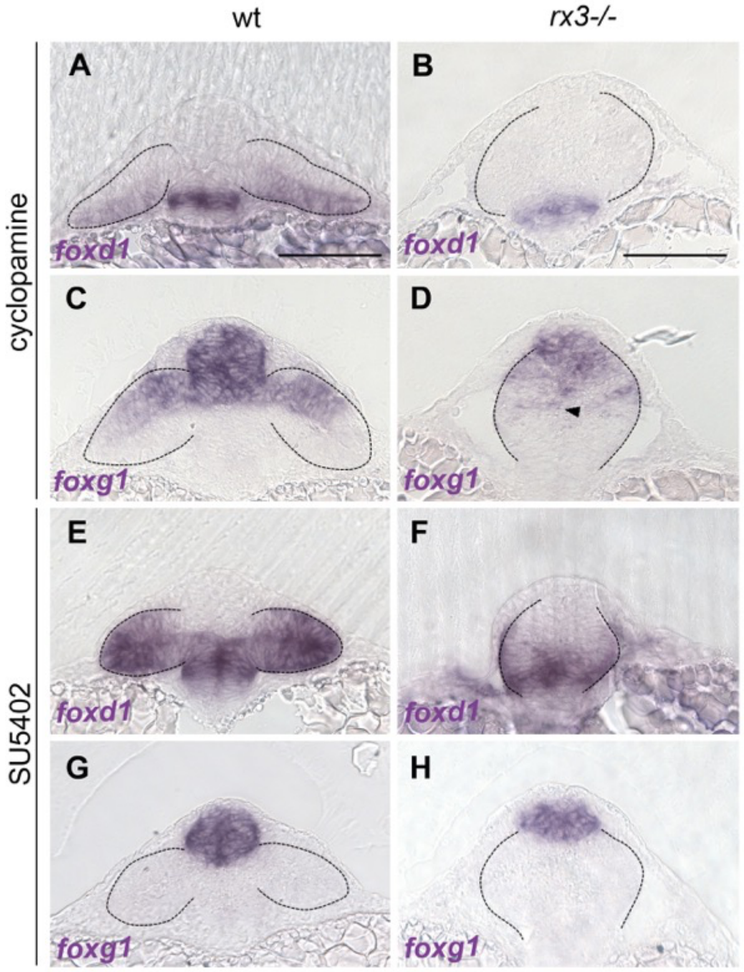

Fig. S1. abrogation of Shh or Fgf activity in *rx3* mutant embryos.
Frontal sections at the level of the forming telencephalon, optic vesicles and hypothalamus with dorsal up. Genes analysed and genotypes are shown bottom left and top respectively.
(A-D) Cyclopamine treated 10ss *rx3*$^{-/-}$ (B,D) and sibling embryos (A,C).
(E-H) SU5402 treated 10ss *rx3*$^{-/-}$ (F,H) and sibling embryos (E,G).

Development • Supplementary information

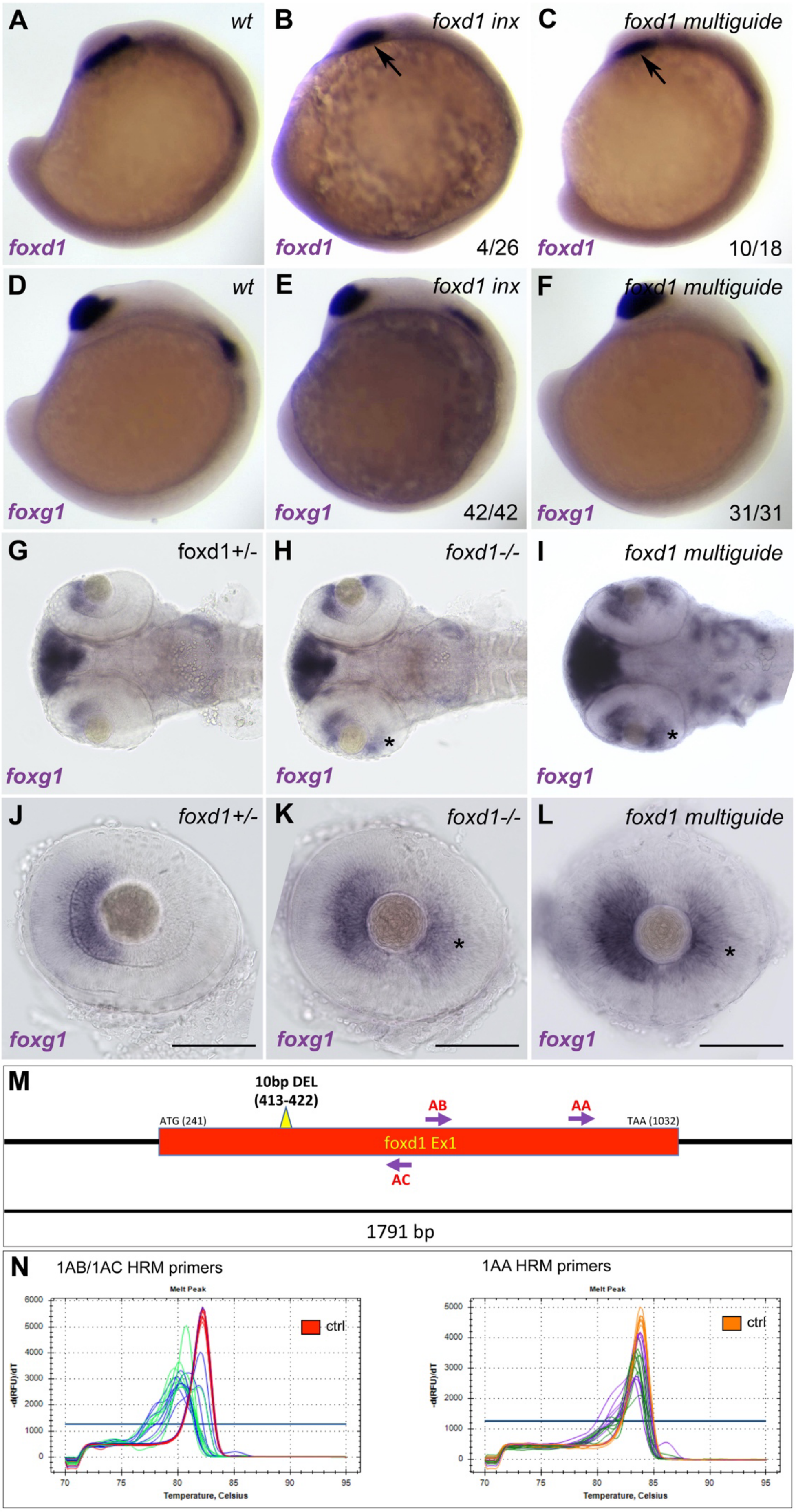

Development • Supplementary information

Fig. S3. *foxd1* "crispants" reproduce the *foxg1* expression expansion phenotype observed in *foxd1* mutants. (A-F) Lateral views of 12-14hpf wild type (A,D), *foxd1*$^{-/-}$ mutants (B,E) and *foxd1* crispants (C,F) labelled with *foxd1* (A-C) or *foxg1* (D-F). Note the reduced *foxd1* domain in *foxd1* mutants (arrow, B) and crispants (arrow, C). (G-L) dorsal views of brains and eyes (G-I) and lateral views of eyes (J-L) showing *foxg1* expression in 60-72hpf wildtype (G,J), *foxd1*$^{-/-}$ mutants (H,K) and *foxd1* crispants (I,L). Note the expansion of *foxg1* to the temporal retina (asterisk in H-I,K-L). (M) Schematic of the *foxd1* locus with the regions targeted by the CRISPR guides highlighted. (N) HRM profile in crispant embryos as compared to the wildtype profile. Two sets of primers were used to assess *indels* induced by guide 1AA (right panel) and guides 1AB/1AC (left panel). The red profile on the left panel and orange profile on the right panel correspond to wildtype controls. All genotyped embryos showed *indels* in both regions.

Development • Supplementary information

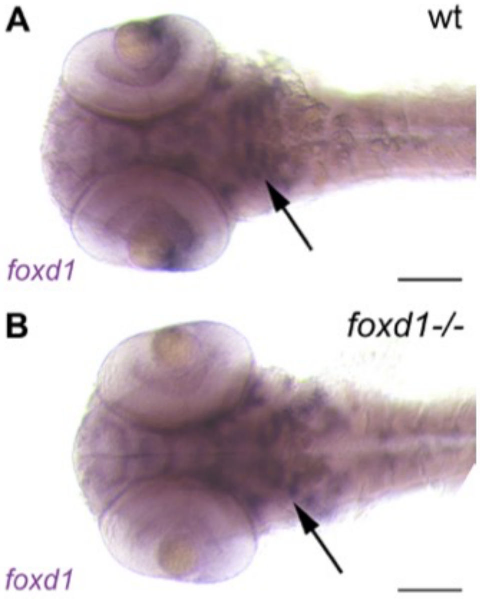

Fig. S4. loss of *foxd1* expression in the temporal retina of *foxd1* mutants is not due to nonsense-mediated RNA decay. Ventral views of heads showing *foxg1* expression in 60-72hpf wildtype (A) and *foxd1*$^{-/-}$ mutants (B). Note *foxd1* expression in the brain and branchial arches regions (arrows) is maintained in *foxd1*$^{-/-}$ mutants (B, compare to A), despite being absent in the retina. Anterior is to the left.

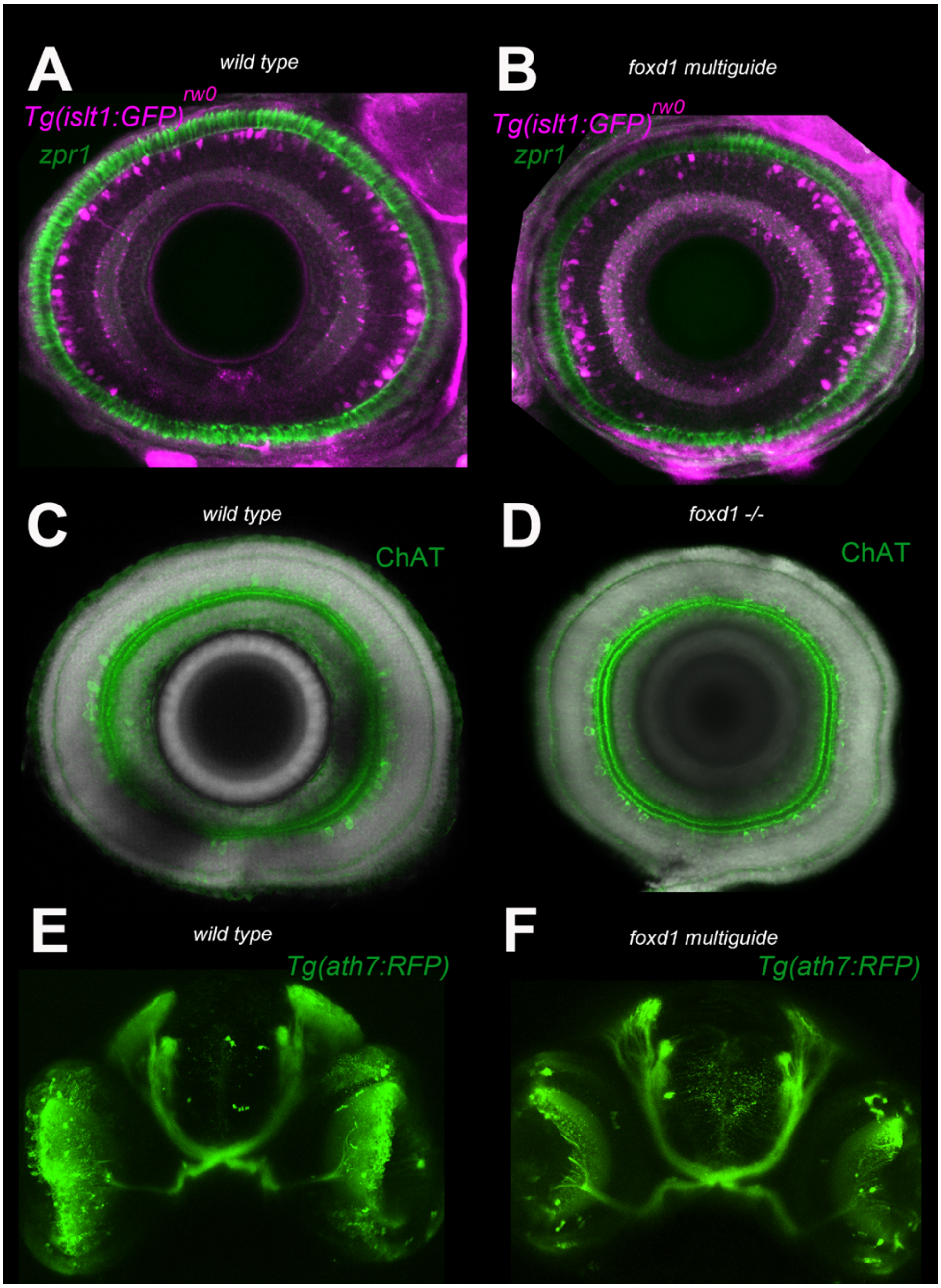

Fig. S5. retinal architecture and chiasma integrity are normal in the loss of *foxd1*. (A-B) Sagittal section across a wildtype (A) and *foxd1* crispant (B) eye immunostained with anti-zpr1 (red and green cones, green) and anti-GFP (amacrine and retinal ganglion cells, (*Tg(isl1:GFP),* magenta) in 8 dpf larvae. (C-D) Immunostaining with anti-Choline Acetyltransferase (ChAT, green) and DAPI (grey) in 7 dpf wild types (C) and *foxd1*$^{-/-}$ mutants (D). (E-F) Frontal view of wild type (E) and *foxd1* crispant (F) 4 dpf larvae highlighting RGC projections (*tg(atoh7:GFP)*, green). All crispant embryos analysed carry *indels* as confirmed by HRM analysis (not shown).

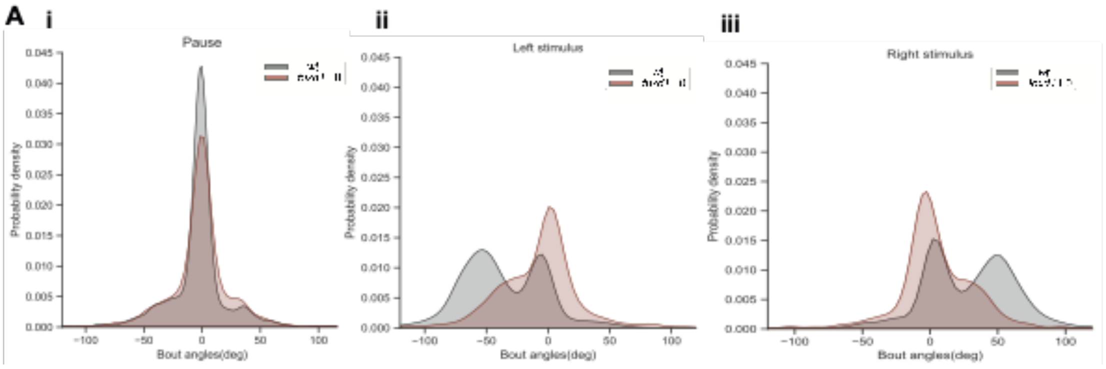

Fig. S6. range of swim bout angles is similar in wildtype and *foxd1* larvae. Probability densities of swim bout angles for wildtype and *foxd1* crispant larvae, during the pause interval (i), the leftwards (ii) and rightwards (iii) oriented whole field motion stimulus.

Table S1. ***Crispr guides and genotyping primers***

Primers used	Sequence
foxd1 original CRISPR guide	GCTTGTAGGGGTCCCGTGC
foxd1 original CRISPR-STAT-fwd	TGTAAAACGACGGCCAGTTCAGATGCACGACGAGATC
foxd1 original CRISPR-STAT-rev	GTGTCTTGTCACAAATCTCGCTCAGC
foxd1 original CRISPR HRM-fwd	CAGATGCACGACGAGATCCT
foxd1 original CRISPR HRM-rev	GAAGTCACAAATCTCGCTCAGC
foxd1.1AA guide	GGCTATGGACCCTACGGTTG
foxd1.1AB guide	TCAAGATACCACGAGAGCCC
foxd1.1AC guide	CGGATAGAGTTCTGCCAAGC
foxd1-1AB/1AC HRM-fwd	GCTGAGCGAGATTTGTGACTTC
foxd1-1AB/1AC HRM-rev	CTAGCGTCCAGTAGTTGCCTTTG
foxd1-1AA HRM-fwd	GCGAGCACGGAGGTTTTCTTCC
foxd1-1AA HRM-rev	GAAAGGCGAGGAGCGCGGAATG

Development • Supplementary information

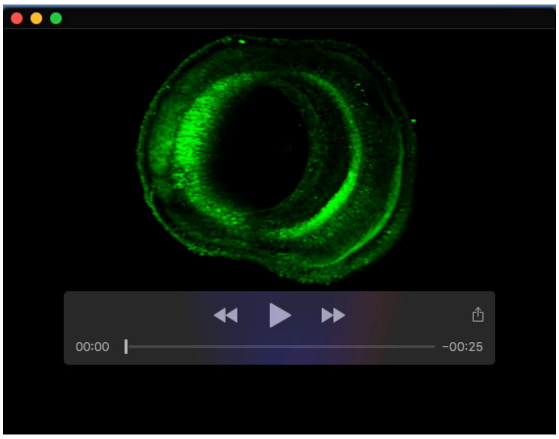

Movie 1. 3D Imaging of a 7dpf wildtype eye immunostained with anti-*PKCa* antibody (green). All movies are oriented with nasal to the left and dorsal up.

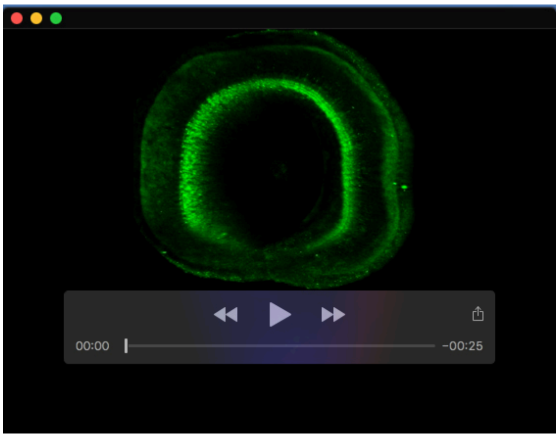

Movie 2. 3D Imaging of a 7dpf *foxd1*$^{-/-}$ mutant eye immunostained with anti-*PKCa* antibody (green).

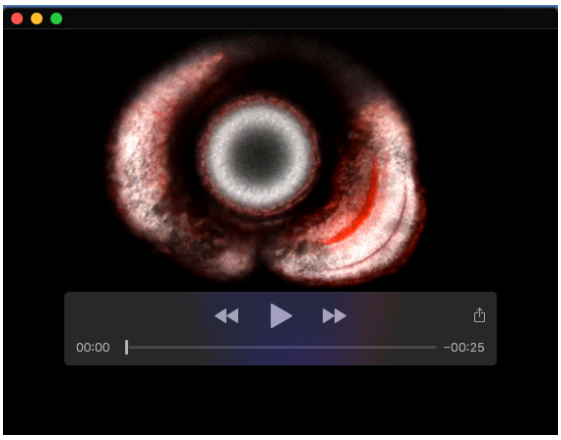

Movie 3. z-stack of a 7dpf wildtype eye immunostained with anti-ChAT (red) and counterstained with DAPI (grey).

Development • Supplementary information

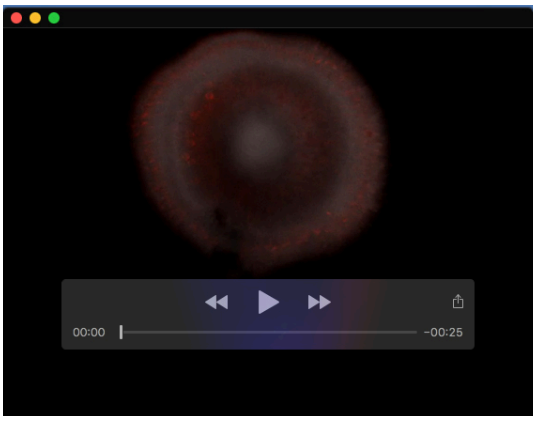

Movie 4. z-stack of a 7dpf *foxd1*$^{-/-}$ mutant eye immunostained with anti-ChAT (red) and counterstained with DAPI (grey).

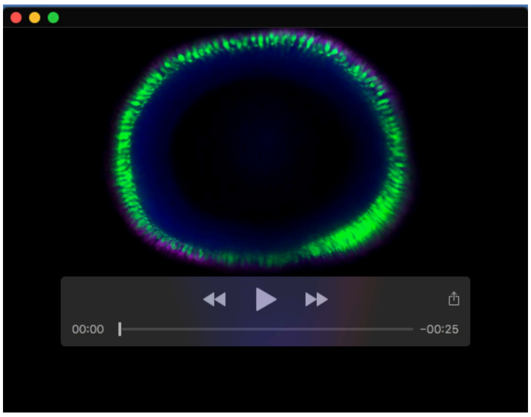

Movie 5. z-stack of a 8dpf *Tg(opn1sw1:GFP),* wildtype eye immunostained with anti-GFP (UV cones, green), anti-zpr1 (green and red cones, magenta) and counterstained with DAPI (blue).

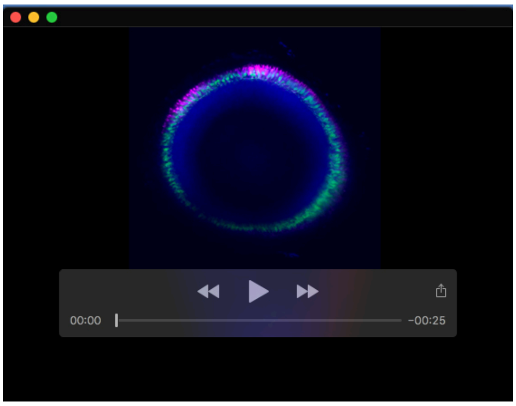

Movie 6. z-stacks of a 8dpf *Tg(opn1sw1:GFP), foxd1* crispant eye immunostained with anti-GFP (UV cones, green), anti-zpr1 (green and red cones, magenta) and counterstained with DAPI (blue).

Development • Supplementary information
