# Supplementary material for: Foxd1-dependent induction of a temporal retinal character is required for visual function
Source: Development. 2022 Dec 19;149(24):dev200938. doi: 10.1242/dev.200938 (PMC9845753; doi:10.1242/dev.200938)
Supplement: Supplementary file 1 [file develop-149-200938_review_history.pdf]

# Foxd1-dependent induction of temporal retinal character is required for visual function

María Hernández-Bejarano, Gaia Gestri, Clinton Monfries, Lisa Tucker, Elena I. Dragomir, Isaac H. Bianco, Paola Bovolenta, Stephen W. Wilson and Florencia Cavodeassi
DOI: 10.1242/dev.200938

**Editor**: François Guillemot

**Review timeline**
Original submission: 12 May 2022
Editorial decision: 14 June 2022
First revision received: 17 October 2022
Accepted: 14 November 2022

---

**Original submission**

First decision letter

MS ID#: DEVELOP/2022/200938

MS TITLE: Foxd1 dependent induction of temporal retinal character is required for visual function

AUTHORS: Maria Hernandez Bejarano, Gaia Gestri, Clinton Monfries, Lisa Tucker, Elena I Dragomir, Isaac H Bianco, Paola Bovolenta, Stephen W Wilson, and Florencia Cavodeassi

I have now received the reports of three referees on your manuscript and I have reached a decision. The referees' comments are appended below, or you can access them online: please go to BenchPressand click on the 'Manuscripts with Decisions' queue in the Author Area.

As you will see, all the referees express great interest in your work, but they also have significant criticisms and recommend a substantial revision of your manuscript before we can consider publication. In particular, they request that you further characterise the High Acuity Area in Foxd1 mutants; they also ask that you address how direct or indirect is the regulation of Foxd1 and Foxg1 expression by Rx3; Reviewer 3 also recommends that you characterise further the retinal defects in Foxd1 mutant fish.

If you are able to revise the manuscript along the lines suggested, which may involve further experiments, I will be happy receive a revised version of the manuscript. Your revised paper will be re-reviewed by one or more of the original referees, and acceptance of your manuscript will depend on your addressing satisfactorily the reviewers' major concerns. Please also note that Development will normally permit only one round of major revision. If it would be helpful, you are welcome to contact us to discuss your revision in greater detail. Please send us a point-by-point response indicating your plans for addressing the refereeâ€™s comments, and we will look over this and provide further guidance.

Please attend to all of the reviewers' comments and ensure that you clearly highlight all changes made in the revised manuscript. Please avoid using 'Tracked changes' in Word files as these are lost in PDF conversion. I should be grateful if you would also provide a point-by-point response detailing how you have dealt with the points raised by the reviewers in the 'Response to Reviewers' box. If you do not agree with any of their criticisms or suggestions please explain clearly why this is so.

© 2022. Published by The Company of Biologists under the terms of the Creative Commons Attribution License (https://creativecommons.org/licenses/by/4.0/).

Reviewer 1

*Advance summary and potential significance to field*

In this work, Hernández-Bejarano et al have described a mutant foxd1 allele in zebrafish. Following previous work by the group on the naso-temporal regionalization of the retina in zebrafish (Hernández-Bejarano et al 2015), they have characterized now the regulation of foxd1 by rx3, and have analysed further its important role in naso-temporal patterning of the retina. Importantly, they performed functional OKR and OMR assays in the mutants, an aspect that could not be tackled previously in mammalian models. Although Foxd1 loss-of-function alleles have been reported in mice, these animals did not survive after birth due to severe renal defects. The absence of conditional alleles has limited studies exploring visual function in mice mutant models for this gene.

Therefore, this is not only a relevant and novel study confirming foxd1 role in naso-temporal patterning in teleosts, it also presents a new vertebrate model that may allow exploring the link between naso-temporal specification and visual function. Being said that, there are several aspects of the work that need to be improved before publication (see below).

*Comments for the author*

Main comments.
a) In Figure 1 the authors show compelling evidence that Rx3 regulates foxd1 and foxg1 expression. However, it is unclear how direct is this regulation, as its precise molecular mechanism is not addressed in the study. It is known that Rx3 acts as a pioneer factor within the complex regulatory network that specifies the neural retina (including proteins encoded by the rx, vsx, hmx, lhx, six, and pax gene families), and thus its mutation has been reported to weaken the retinal network. The authors should discuss to which extent Rx3 could be directly responsible to control N-T identity, or alternatively if the abnormal retinal patterning emerges from the reduced activity of the entire network.

b) To confirm previous overexpression results showing foxd1/foxg1 cross-repression (Hernández-Bejarano et al 2015) and to evaluate the impact of foxd1 loos of function in early NT patterning, an assessment of foxd1 and foxg1 levels in 10 ss foxd1-/- embryos should be provided.

c) PKCa staining is used as the only feature defining the size and architecture of the HAA in this study. A better characterization of this area (based on the cones/rods ratio and density), would help to understand the relationship between NT patterning and HAA differentiation. Also, given the subtle changes, the authors should provide a quantification and statistical analysis of the differences observed between wt, foxd1-/- and Tg{rx3:Gal4};UAS:Shh retinas in PKCa immunostainings (figure 2N-P).

d) My main concerns with the study have to do with the assessment of the visual defects in foxd1-/- and Tg{rx3:Gal4};UAS:Shh models at 6dpf (Figure 3). The authors claim that the results of these experiments, particularly the normal OKR in Tg{rx3:Gal4};UAS:Shh, suggest a prominent role of the temporal retina in controlling visual function. Several aspects need to be improved/explored further here:
- Although the statistical test used to determine significance are described in the text (ANOVA double test, followed by a Bonferroni test) the p value is not indicated in any of the panels in figure 3. This information needs to be incorporated in each panel, particularly when significant differences were found.
- While abnormal OKR and OMR responses are a general readout of altered visual function, they are insufficient to determine at which level is the visual pathway defective. On the light of previous studies, the abnormal retino-tectal projection could be hypothesised as the ultimate cause for the visual defects. However, both foxd1 and foxg1 have a complex expression pattern during development and alternative explanations cannot be ruled out prematurely. Particularly, it will be important to test whether retinal lamination is normal in 6 dpf mutant retinas (a standard DAPI/phalloidin staining would be sufficient), whether the retinal activity (i.e. as recorded by ERG) is normal, and whether the chiasmatic region is properly specified in the mutants. Regarding this

© 2022. Published by The Company of Biologists under the terms of the Creative Commons Attribution License (https://creativecommons.org/licenses/by/4.0/).

last aspect, a Foxd1 requirement for proper formation of the optic chiasm has been demonstrated in mice (Herrera et al 2004).
- Finally, if abnormal retino-tectal projections are the ultimate cause of the impaired visual function, the result showing that the normal OKR response in Tg{rx3:Gal4};UAS:Shh animals is unexpected: given the severely altered retino-tectal projection previously described for this model (Hernández-Bejarano et al 2015). To explain this, the authors indicate that the phenotypic discrepancy between foxd1-/- and Tg{rx3:Gal4};UAS:Shh models may be due to the nasal retina not contributing to the OKR (a conclusion even hinted in the abstract). However, I feel this aspect has not been sufficiently addressed in the work, as the central visual dominance recently described in Dehmelt et al 2021 for OKR seems a centro-peripheral graded response rather than an all-or-none property. The fact that the Tg{rx3:Gal4};UAS:Shh model is not a stable transgenic line but depends on the injection of the GFP:UAS:shh construct in one-cell Tg{rx3:Gal4} embryos is also concerning in this regard. Although embryos with homogeneous GFP expression in the retina have been selected for the OKR assays, the integrity of their retino-tectal projections was not examined for the same larvae.

Taking all these comments into consideration, the conclusion that temporal retina plays a prominent role in visual function needs to be revisited. One possibility is bringing additional evidence explaining at which level is the visual pathway impaired in foxd1 mutants. Alternatively, this (premature) conclusion can be removed from the article, which independently of this particular aspect includes enough valuable data to be considered for publication.

Additional Minor comments.

-Figure 1-I shows the double ISH foxg1/nkx2.1; foxd1/nkx2.1 for rx3 mutant. If available, it would be informative including additional comparative panels showing these markers in wild type embryos.
-The protein is refereed through the text as FoxD1. However, I think the consensus symbol should be Foxd1.
-Figure S4 is called before S3. They should be renamed to keep the order.
- The low resolution in Figure S5 does not allow distinguishing legends and axes labelling.
- In the scheme in Fig 3A the retinotectal projections from the Tg{rx3:Gal4};UAS:Shh retinas do not correspond to the previous description in (Hernández-Bejarano et al 2015). In that study nasal projections invaded the whole tectum, whereas in 3A scheme only the anterior region appears as a target.

Reviewer 2

*Advance summary and potential significance to field*

The authors of this paper demonstrate the necessity of Rx3 and FoxD1 in the establishment of the High Acuity Area (HAA) in Zebrafish. ItÂ’s been shown that knockout of Rx3 leads to a severe disruption of Optic Vesicle (OV) development as well as a loss of FoxD1 expression. Interestingly this reduction was not rescued when the embryos were treated with Shh signaling inhibitor Cyclopamine and FGF signaling inhibitor SU5402. The authors next generated a loss-of-function mutant of FoxD1 to assess its role during the establishment of HAA.
They confirmed that this mutant also exhibited naso-temporal axial defects that led to improper axon projection of the Retinal Ganglion Cell (RGC). In addition the localization of the HAA in these mutants was disrupted. Finally, they associated this disruption with the oculomotor and optokinetic response defects.

*Comments for the author*

While the manuscript is well written and the experiments were well performed and clearly presented, the major concern is the lack of novelty and scientific significance of these results. More specifically, the authors demonstrated the necessity of Rx3 for the proper establishment of the FoxG1 and FoxD1 expression boundaries of the OV but did not further investigate the mechanism regarding this phenotype in vivo, as it could be an indirect result due to the severe disruption of OV development (Stigloher 2006). While the failure of SU5402 treatment to rescue the

© 2022. Published by The Company of Biologists under the terms of the Creative Commons Attribution License (https://creativecommons.org/licenses/by/4.0/).

expression of FoxD1 is an interesting finding, the requirement of Rx3 for FoxD1 expression has been shown in zebrafish as cited by the authors (Yin 2014). Similarly, the characterization of Ephrins in the FoxD1 mutants is elegantly presented, but the role of FoxD1 in the naso-temporal patterning of the retina has also been well studied previously (Carreres 2011).
Finally, the requirement of FoxD1 for the establishment of HAA, while novel, has not been sufficiently characterized. Importantly, the only evidence for the HAA defect is the subtle reduction in PKC-a expression, which is obscured by uneven exposure and staining as indicated by the DAPI staining. It is important to test other HAA characteristics in these mutants such as the density of the rod photoreceptors as well.

Minor comments:
1. The authors claim that FoxG1 expression is expanded throughout most of the prospective eye domain but there is no clear regional delineation in their staining, a co-staining with a pan OV marker such as Six3 could strengthen their argument.

2. It would be nice to demonstrate that the DiO-labelled axons of the FoxD1 mutants project throughout the optic track by showing a wholemount picture of these embryos.

3. The number of embryos examined and the penetrance of mutant phenotypes should be indicated in Figures 1 and 2.

Reviewer 3

*Advance summary and potential significance to field*

Hernandez-Bejarano and colleagues have extended their prior work using the zebrafish model system to elucidate further mechanistic insights into the interplay of sonic hedgehog (SHH) and fibroblast growth factor (FGF) signalling and the forkhead transcription factor foxd1 towards understanding temporal/nasal patterning and visual system function. Of interest, this interplay requires the rx3 transcription factor. The authors incorporate loss- and gain-of function assays in vivo through the use of contemporary methods to substantiate their work. This work further supports the area for high acuity vision (HAA) in the zebrafish as similar to the fovea in humans and other vertebrates (birds, primates).

*Comments for the author*

Major Concerns:
1. Results, Figure 1: The authors should present the data for Cyclopamine and SU5042 separately, as well as combined.
2. Results, Figure 2: The use of only one marker, PKCalpha, is not sufficient. At least one additional marker is recommended for assessment of the HAA in the zebrafish.
3. For the foxd1 mutants, there was very limited characterization of the developing or mature retina. For example, there was no assessment of cell-type specific markers for the 6 neuronal and one glial retinal cell type. This presents a missed opportunity to understand the role of the foxd1 transcription factor in retinal development and function.

Specific Concerns:
1. Results, page 4, lines 122-127: This paragraph should be moved into the Introduction.
2. The rx3-/- mutant requires explanation in either the Methods or first use in the Results section.
3. How were the doses of Cyclopamine and SU5042 determined? Only one dose was used per specific treatment.
4. Although a stop codon is predicted for the foxd1 mutant, the authors haven't established (using assays such as Western blotting) that the protein is actually truncated.
5. No specific examples of retinotectal projection data was shown; only a summary of pooled data in Figure 3. What was the duration of labelling with DiI and DiO?
6. The Discussion could be expanded to mention limitations of the OKR and OMR visual function tests and what other tests could be performed using other model systems.

© 2022. Published by The Company of Biologists under the terms of the Creative Commons Attribution License (https://creativecommons.org/licenses/by/4.0/).

Other Concerns:
1. Abstract: Bring the term "fovea" into the abstract.

---

**First revision**

Author response to reviewers' comments

We would like to thank the reviewers and you for the comments on our manuscript. We are glad to read that "*this is not only a relevant and novel study confirming* foxd1 *role in naso- temporal patterning in teleosts, it also presents a new vertebrate model that may allow exploring the link between naso-temporal specification and visual function*" and appreciate the many positive comments from the reviewers. We acknowledge that there are also several areas for improvement. Below, we provide a point-by-point answer to the comments from the reviewers, highlighting the areas in the manuscript that have been updated to reflect new results or discussion points.

**Reviewer 1 comments:**

We thank the reviewer for their encouraging comments. We are glad that the reviewer appreciates the relevance of our study (*"…this is not only a relevant and novel study confirming foxd1 role in naso-temporal patterning in teleosts, it also presents a new vertebrate model that may allow exploring the link between naso-temporal specification and visual function."*) and the additional analysis avenues this new model opens ("*Importantly, they performed functional OKR and OMR assays in the mutants, an aspect that could notbe tackled previously in mammalian models.*"). The reviewer also highlights several aspects that should be improved and we provide our answers to those points below.

Main comments.

**1a)** *In Figure 1 the authors show compelling evidence that Rx3 regulates foxd1 and foxg1 expression. However, it is unclear how direct is this regulation, as its precise molecular mechanism is not addressed in the study. It is known that Rx3 acts as a pioneer factor within the complex regulatory network that specifies the neural retina (including proteins encoded by the rx, vsx, hmx, lhx, six, and pax gene families), and thus its mutation has been reported to weaken the retinal network. The authors should discuss to which extent Rx3 could be directly responsible to control N-T identity, or alternatively if the abnormal retinal patterning emerges from the reduced activity of the entire network.*

As the reviewer correctly points out, our results do not allow us to determine whether *foxd1*/*foxg1* expression is directly regulated by Rx3. Our results only show that *foxd1* expression is lost in the anterior neural plate of *rx3* mutants. In agreement with our observations, previous transcriptomic data (Yin et al., 2014, BMC Genomics) identified *foxd1* as one in a list of strongly downregulated genes in *rx3* mutants. That study further identified potential *rx3* binding sites in the *foxd1* promoter, suggesting a direct regulation of *foxd1* expression by Rx3. However, the functionality of these binding sites was not demonstrated and thus it is unclear whether they are relevant for the control of *foxd1* expression.

The reviewer acknowledges that Rx3 is a central factor in the complex gene network controlling eye fate. Removing *rx3* function leads to deregulation in the expression of many of the other eye-field specification transcription factors, resulting in the eventual loss of retinal identity. We thus cannot discard the possibility that the loss of *foxd1* expression is due to the loss of retinal identity, downstream of the overall mis-regulation of expression of eye-field specification transcription factors.

To try to provide more specific data regarding the control of *foxd1* by *rx3*, we generated transplants of *rx3* mutant cells into wild type embryos, and vice-versa. Our rationale is that if *rx3* directly controls *foxd1*, local loss of *rx3* in the ventral optic vesicle would result in an

© 2022. Published by The Company of Biologists under the terms of the Creative Commons Attribution License (https://creativecommons.org/licenses/by/4.0/). 

autonomous loss of *foxd1*; conversely, a group of wild type cells in the ventral eye field of *rx3* mutants would express *foxd1* autonomously.

The results of this experiment, however, were not conclusive. *rx3* mutant cells became systematically positioned in the dorsal portion of the anterior neural keel in wild type hosts, excluded from the optic vesicle (Rebuttal Figure 1B), underscoring the requirement of *rx3* expression to adopt retinal identity. This precluded us from analysing the effect of this manipulation on *foxd1* expression. Very few transplants were recovered from the opposite condition (wild type cells into *rx3* mutant hosts); when big enough, these transplants generated an “eyelet” that attempts to evaginate (Rebuttal Figure 1C). Only a subset of the transplanted cells showed expression of *foxd1*; we interpret this would become the ventral part of the ectopic eyelet.

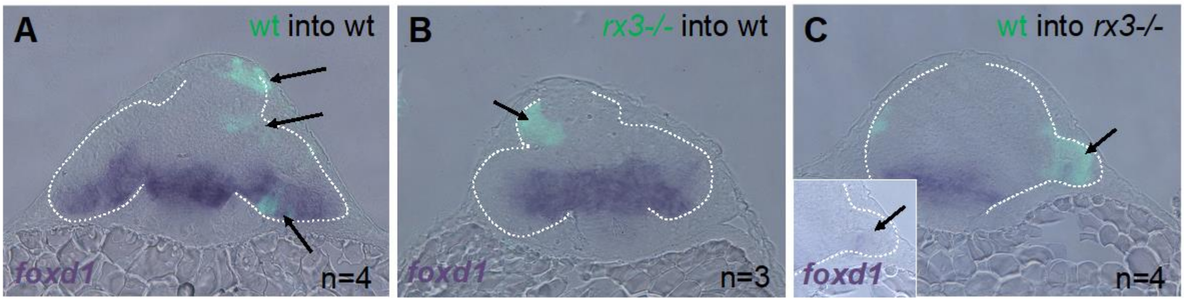


**Rebuttal Figure 1**: Transplants (green) of wildtype cells into wildtype embryos (A), *rx3-/-* into wildtype (B) and wildtype into *rx3-/-* (C). While wild type transplants into a wild type background distribute throughout the anterior neural keel (A, arrows), *rx3-/-* transplants into wild type form tight clumps that segregate outside of the optic vesicle (B, arrow). Wild type transplants into *rx3-/-* generate an eyelet (C, arrow) and are associated with expression of *foxd1* (purple) in the ventral region of the evaginating eyelet (inset in C). All panels show frontal sections through the anterior neural keel. “n” corresponds to the samples that have been sectioned and analysed in detail.

Since the numbers of transplants we have recovered is limited, and the results not conclusive, we prefer not to present these results in the manuscript. Nevertheless, and following the reviewer’s suggestion, we have included in the manuscript (page 9; lines 329-335) a more elaborated discussion of possible modes of *foxd1* regulation by Rx3 (direct/indirect) explicitly stating that we cannot discard either possibility.

***1b)*** *To confirm previous overexpression results showing foxd1/foxg1 cross-repression (Hernández-Bejarano et al 2015) and to evaluate the impact of foxd1 loss of function in early NT patterning, an assessment of foxd1 and foxg1 levels in 10 ss foxd1-/- embryos should be provided.*

We agree with the reviewer on the importance of determining the impact of the loss of *foxd1* on *foxg1* and *foxd1* expression. We have undertaken this analysis, and we present these new data below (Rebuttal Figure 2). We hope the reviewer will agree with us that they provide now a more complete picture of the effect of the loss of *foxd1* on *foxd1/foxg1* expression.

*foxd1* expression shows a variable pattern in *foxd1* mutants and multiguide injected embryos (crispants) at early stages. A subset of 12-14hpf embryos derived from the incross of *foxd1* heterozygote parents show a moderate reduction in *foxd1* expression (Rebuttal Figure 2A-C; 4 out of 26 embryos), which is reproduced in the crispants (Rebuttal Figure 2D-E; 10 out of 18 embryos). Expression of *foxg1* is however normal in both mutants and crispants at 12- 14hpf (Rebuttal Figure 2F-H; 42 and 31 embryos analysed, respectively). Ectopic expression of *foxg1* in the temporal half of mutant retinae can only be detected from 24hpf onwards (Rebuttal Figure 2I-P; genotype of embryos confirmed by HRM analysis).

© 2022. Published by The Company of Biologists under the terms of the Creative Commons Attribution License (https://creativecommons.org/licenses/by/4.0/).

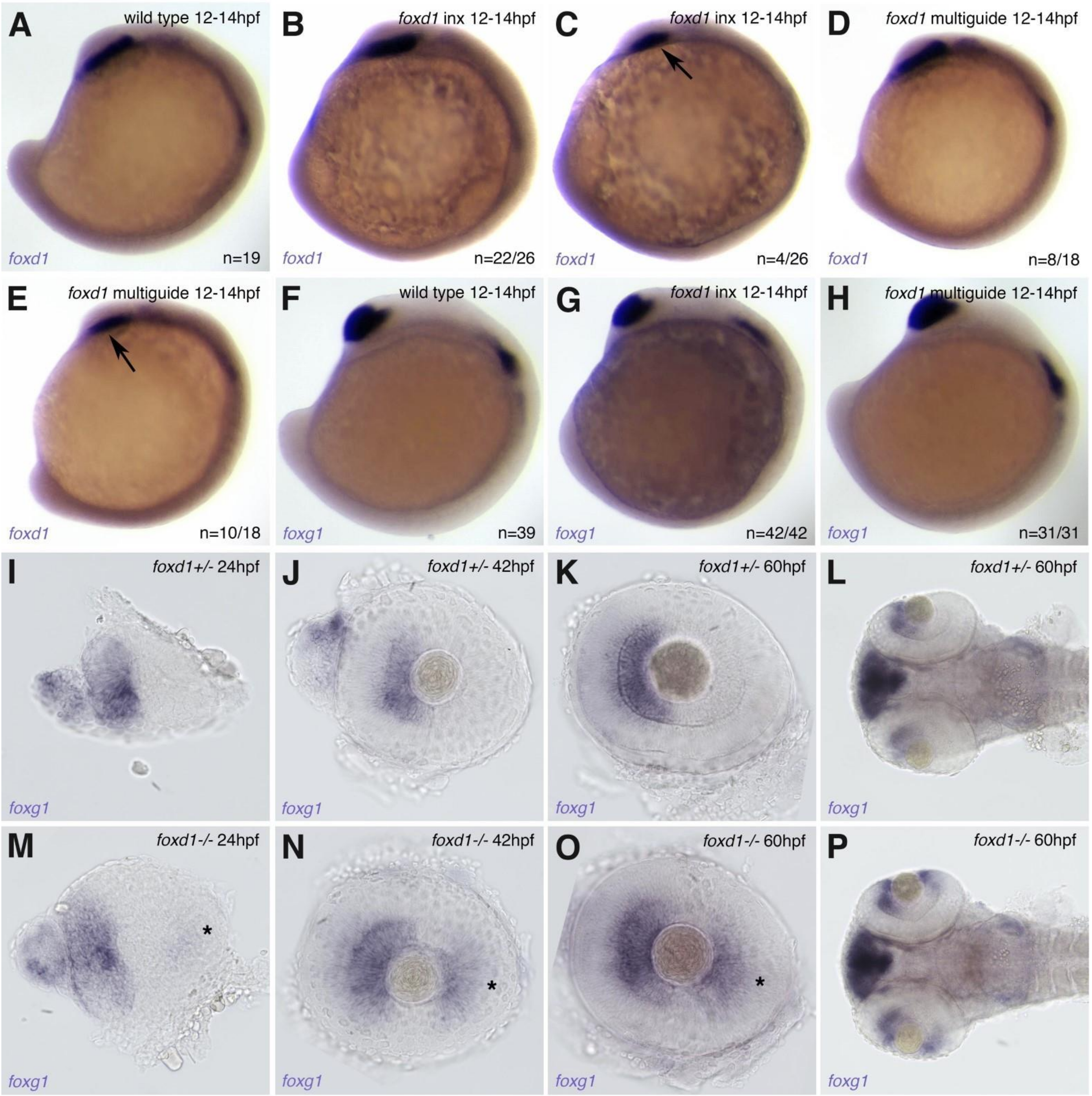


**Rebuttal Figure 2**: *foxd1*/*foxg1* expression is affected in *foxd1* mutants and crispants from 24hpf onwards. (A-H) Lateral views of 12-14hpf embryos labelled with *foxd1* (A-E) or *foxg1* (F-H). Arrows in (C,E) highlight a reduced *foxd1* domain. (I-P) Expression of *foxg1* in the optic cup of *foxd1* mutants (M-P) and siblings (I-L). Asterisks in (M-O) highlight the ectopic expression of *foxg1* in the temporal retina. (I-K, M-O) are lateral views of dissected eyes; (L,P) are dorsal views of whole heads. All images are oriented with anterior to the left. Genotype of the embryos, stage analysed and marker used are detailed in the corresponding panel.

The fact that changes in *foxg1* expression in *foxd1* mutants are only detected from 24hpf onwards may not be so surprising. The upstream signals controlling the initial establishment of this pattern are likely to be intact in the mutants (see Supplementary Figure 1 in the manuscript), and thus it would be expected that initial nasotemporal regionalisation happens normally. Our interpretation of these results is that, since there is no functional Foxd1 in the mutants, refinement of the *foxd1*/*foxg1* boundary by cross-repression does not occur and eventually *foxg1* expands into the temporal half of the eye. These results complement those presented in Figure S3 in the original manuscript.

© 2022. Published by The Company of Biologists under the terms of the Creative Commons Attribution License (https://creativecommons.org/licenses/by/4.0/).

We have now incorporated this information in page 6, lines 197-206. Figure S3 has been expanded to include these data.

***1c)*** *PKCa staining is used as the only feature defining the size and architecture of the HAA in this study. A better characterization of this area (based on the cones/rods ratio and density), would help to understand the relationship between NT patterning and HAA differentiation.*

We agree with the reviewer that a more extensive characterisation of the HAA will strengthen our interpretations. We present below the results of analysing the distribution and morphology of UV cones. UV cone morphology varies across the zebrafish retina, with those present in the HAA region ten-times longer than UV cones elsewhere in the retina (Yoshimatsu et al., Neuron 107, 320-337, 2020).

Imaging UV cones morphology in *tg(opn1sw1:GFP)* fish highlights a qualitative difference in the appearance of the HAA region in *foxd1* crispants as compared to wild types (Rebuttal Figure 3A-B). Quantification of UV cone length in these animals confirms that UV cones in the HAA are significantly shorter in *foxd1* crispants as compared to wild-type embryos, and overall similar in length to UV cones in the nasal retina (Rebuttal Figure 3C).

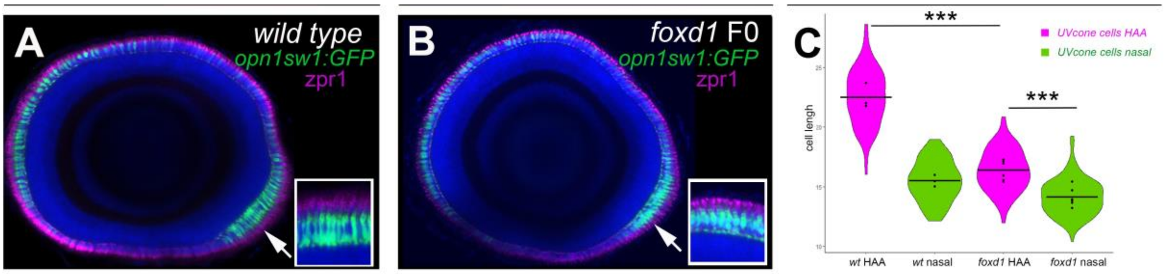


**Rebuttal Figure 3:** (A-B) Sagittal sections across *wildtype* (A) and *foxd1* crispant (B) eyes with nuclei stained by DAPI (blue), the outer photoreceptor segments stained by zpr1 (magenta) and UV cones expressing GFP (green, *Tg(opn1sw1:GFP)*) in 8 dpf larvae. (B) presents an example of the milder phenotype. (C) Violin plots showing differences in cone cell length between cells located in the HAA and nasal retina in *wildtype* and *foxd1* crispants. Mean difference in length of 7.003 pixels between wildtype HAA and nasal cells (95% family-wise confidence interval: 6.15–7.855, $p < 0.001$; n =78 cells each for wildtype HAA and nasal regions from n=3 eyes) and 2.250 pixels in *foxd1* (95% CI: 1.771–2.729, $p < 0.001$, 156 cells each for foxd1 crispant regions from n=6 eyes). One- way analysis of means, not assuming equal variances (F = 279.35, num df = 3.00, denom df = 200.85, p-value < 2.2e-16), followed by Games-Howell *post hoc* testing. Crossbars showing the mean cell length for each condition; dots showing the mean cell lengths for each individual eye.

These results complement those described in the manuscript and strengthen our interpretation of the *foxd1* mutant phenotype. We have now incorporated them in the manuscript (page 6-7, lines 231-248) and in Figure 2Q-S.

*Also, given the subtle changes, the authors should provide a quantification and statistical analysis of the differences observed between wt, foxd1-/- and Tg{rx3:Gal4};UAS:Shh retinas in PKCa immunostainings (figure 2N-P).*

We have attempted to do these quantifications. However, this has proven not trivial to perform, and we have not succeeded in doing the analysis in a way that we consider to be consistent, reproducible and robust. We consider this does not invalidate the clear qualitative differences that we observe in the extent of immunostaining, which are particularly obvious when comparing the wild type with the *foxd1* mutant situation. We have added videos of a 3D reconstruction of PKCa staining in wildtype and *foxd1-/-* eyes as supplementary video 1 (SV1) and supplementary video 2 (SV2) in order to alleviate concerns that wild-type/mutant differences could be due to difference in eye orientation.
Moreover, as described above, we now provide a quantification of changes in length of UV cones, which we hope the reviewers will agree support our interpretation.

© 2022. Published by The Company of Biologists under the terms of the Creative Commons Attribution License (https://creativecommons.org/licenses/by/4.0/). 

**1d)** *My main concerns with the study have to do with the assessment of the visual defects in foxd1-/- and Tg{rx3:Gal4};UAS:Shh models at 6dpf (Figure 3). The authors claim that the results of these experiments, particularly the normal OKR in Tg{rx3:Gal4};UAS:Shh, suggest a prominent role of the temporal retina in controlling visual function. Several aspects need to be improved/explored further here:*

*- Although the statistical test used to determine significance are described in the text (ANOVA double test, followed by a Bonferroni test) the p value is not indicated in any of the panels in figure 3. This information needs to be incorporated in each panel, particularly when significant differences were found.*

We apologise for this omission, due to a last-minute reorganisation of Figure 3. We have now reincorporated this information in the manuscript. Statistical significance is highlighted when present in Figure 3, and the values recovered from the analysis are now included in Page 8, lines 285-309.

*- While abnormal OKR and OMR responses are a general readout of altered visual function, they are insufficient to determine at which level is the visual pathway defective. On the light of previous studies, the abnormal retino-tectal projection could be hypothesised as the ultimate cause for the visual defects. However, both foxd1 and foxg1 have a complex expression pattern during development and alternative explanations cannot be ruled out prematurely. Particularly, it will be important to test whether retinal lamination is normal in 6 dpf mutant retinas (a standard DAPI/phalloidin staining would be sufficient), whether the retinal activity (i.e. as recorded by ERG) is normal, and whether the chiasmatic region is properly specified in the mutants. Regarding this last aspect, a Foxd1 requirement for proper formation of the optic chiasm has been demonstrated in mice (Herrera et al 2004).*

We agree with the reviewer in the fact that our results do not allow us to determine at which level the visual pathway is affected. Our interpretation, based on the fact that gross retinal lamination is not affected (as evident from the images in Figure 2 in the manuscript) and other studies showing that tectal innervation does not impact in OKR performance, was that the loss of temporal fate and HAA features was the most parsimonious explanation for at least part of the defects seen in the mutants.

To strengthen this argument, we have incorporated the following results:

- We have reassessed retinal lamination and differentiation by performing immunostaining against zpr1 (red and green cones), islet1 (amacrine and retinal ganglion cells) and Choline Acetyltransferase (ChAT; starbust amacrine cells). The results, presented below (Rebuttal Figure 4A-D), confirm the overall normal retinal lamination pattern observed in the absence of *foxd1*. We also include z-stacks of DAPI and ChAT in wildtype and *foxd1* mutants as supplementary video 3 (SV3) and supplementary video 4 (SV4) as well as z- stacks of DAPI/Zpr1/ *tg(opn1sw1:GFP)* in both conditions (SV5 and SV6).

We have taken advantage of the *tg(atoh7:GFP)* transgenic line to assess optic nerve projection and chiasma integrity. Our results show that the chiasma is not overtly affected in the absence of *foxd1* (Rebuttal Figure 4E-F). The organisation and number of *tg(atoh7:GFP)* labelled retinal cells is also overall normal when comparing with wild types (not shown).

© 2022. Published by The Company of Biologists under the terms of the Creative Commons Attribution License (https://creativecommons.org/licenses/by/4.0/).

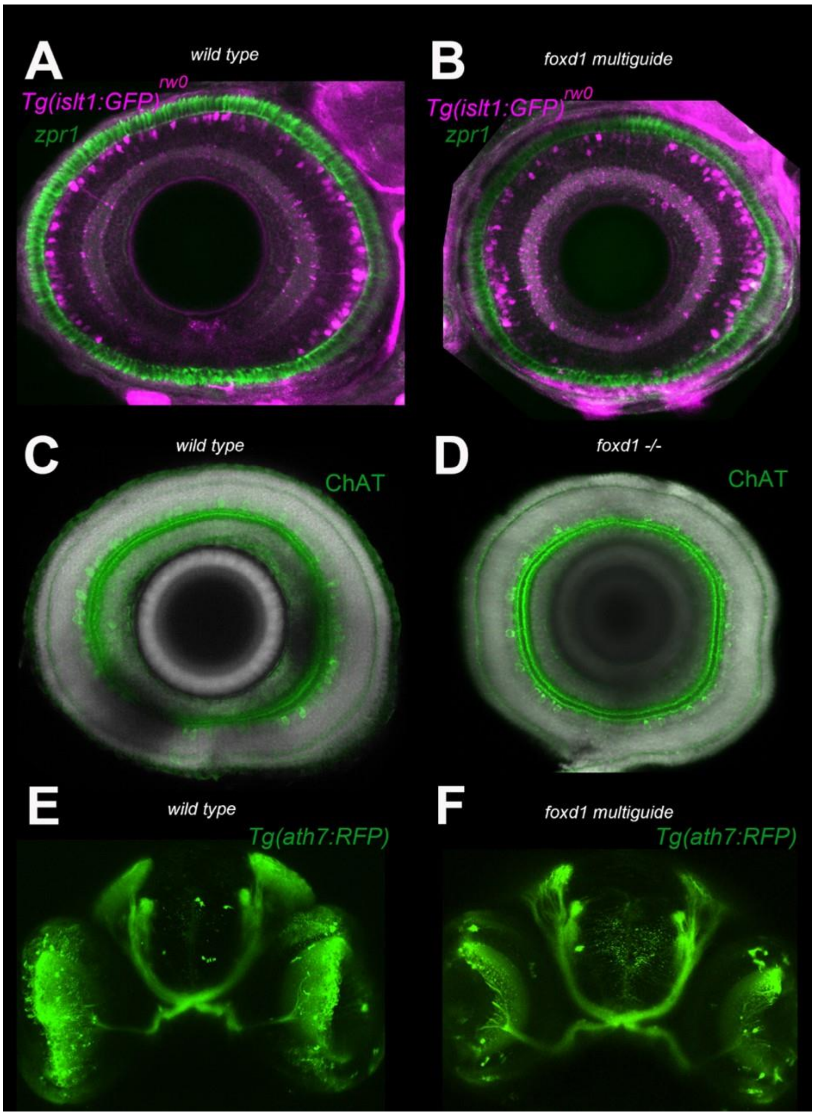


**Rebuttal Figure 4:** (A-B) Sagittal section across a *wildtype* (A) and *foxd1* crispant (B) eye immunostained with anti-zpr1 (red and green cones, green) and amacrine and retinal ganglion cells (*Tg(isl1:GFP),* magenta) in 8 dpf larvae. (C-D) Immunostaining with anti-Choline Acetyltransferase (ChAT, green) and DAPI (grey) in 7 dpf *wildtype* (C) and *foxd1* mutants (D). (E-F) Frontal view of *wildtype* (E) and *foxd1* crispants (F) 4 dpf larvae highlighting RGC projections (*tg(atoh7:GFP)*, green).

Overall these results suggest that retinal differentiation and chiasma integrity are not severely affected in the *foxd1* mutants/morphants. We cannot discard subtle deficiencies, but it seems unlikely that the severity of the visual defects observed could be attributed to those potential subtle defects. We have incorporated these data in the manuscript as a new supplementary figure (New Figure S5, and supplementary videos 3/6) and we describe them in page 7, line 250-255.

*- Finally, if abnormal retino-tectal projections are the ultimate cause of the impaired visual function, the result showing that the normal OKR response in Tg{rx3:Gal4};UAS:Shh animals is unexpected: given the severely altered retino-tectal projection previously described for this model (Hernández-Bejarano et al 2015). To explain this, the authors indicate that the phenotypic discrepancy between foxd1-/- and Tg{rx3:Gal4};UAS:Shh models may be due to the nasal retina not contributing to the OKR (a conclusion even hinted in the abstract).*
*However, I feel this aspect has not been sufficiently addressed in the work, as the central visual dominance recently described in Dehmelt et al 2021 for OKR seems a centro-peripheral graded response rather than an all-or-none property. The fact that the Tg{rx3:Gal4};UAS:Shh model is not a stable transgenic line but depends on the injection of the GFP:UAS:shh construct in one-cell*

© 2022. Published by The Company of Biologists under the terms of the Creative Commons Attribution License (https://creativecommons.org/licenses/by/4.0/). 

*Tg{rx3:Gal4} embryos is also concerning in this regard. Although embryos with homogeneous GFP expression in the retina have been selected for the OKR assays, the integrity of their retino-tectal projections was not examined for the same larvae.*

Indeed, as the reviewer mentions, recent results from Dehmelt et al., 2021 suggest that OKR performance does not depend on a specific region of the retina, and we highlight this apparent discrepancy between their results and our observations in the discussion (Page 9). The reviewer is concerned at the nature of the *Tg(rx3:Gal4)*;UAS:Shh model and argues that this may affect the reproducibility of the results obtained in these animals. However, we would like to argue that despite its limitations, this model is reproducible enough to be used in this context.

We have used this approach in the past (Hernández-Bejarano et al. 2015, Development) and have shown reproducible phenotypes by expression of *foxd1/foxg1* and DiI/DiO tracing. In the current study, we have adopted the same approach to select embryos for analysis and found reproducible changes in the pattern of PKCa expression. Unfortunately, we no longer have the specimens in which the behavioural tests were done and thus we cannot provide information on the retinotectal projections in those samples. In view of this problem, we have toned down our interpretation in the discussion, by modifying the statement on page 9 lines 356-357.

*Taking all these comments into consideration, the conclusion that temporal retina plays a prominent role in visual function needs to be revisited. One possibility is bringing additional evidence explaining at which level is the visual pathway impaired in foxd1 mutants. Alternatively, this (premature) conclusion can be removed from the article, which independently of this particular aspect includes enough valuable data to be considered for publication.*

Following the reviewer's recommendation, we have revisited our analysis and provided additional data where possible (see above), and we have modified the text to reduce the emphasis on the prominent role of the temporal retina.

**1e)** *Additional Minor comments.*

*-Figure 1-I shows the double ISH foxg1/nkx2.1; foxd1/nkx2.1 for rx3 mutant. If available, it would be informative including additional comparative panels showing these markers in wild type embryos.*

Unfortunately, we no longer have these specimens and reproducing similar images for comparison to the ones presented is not possible. However, we would like to argue that adding these images to an already very large Figure 1 will not add much information for the reader, and will make the figure unmanageable. We think a clear comparison can be done from the images already presented, and hope the reviewer will agree with our decision of leaving the figure as it is.

*-The protein is refereed through the text as FoxD1. However, I think the consensus symbol should be Foxd1.*

Thank you for pointing this out. We have now changed the entry to Foxd1 throughout the text.

*-Figure S4 is called before S3. They should be renamed to keep the order.*

In the revised version of the manuscript Figure S3 is called before S4. We have therefore kept the original order.

- *The low resolution in Figure S5 does not allow distinguishing legends and axes labelling.*

We apologise to the reviewer. Our original pdf file has a good resolution and the axes and labels are readable. We hope the re-uploaded figure has the correct resolution.

© 2022. Published by The Company of Biologists under the terms of the Creative Commons Attribution License (https://creativecommons.org/licenses/by/4.0/).

*- In the scheme in Fig 3A the retinotectal projections from the Tg{rx3:Gal4};UAS:Shh retinas do not correspond to the previous description in (Hernández-Bejarano et al 2015). In that study nasal projections invaded the whole tectum, whereas in 3A scheme only the anterior region appears as a target.*

We have reanalysed our description and the images in the 2015 paper, and they seem to match our schematic in Figure 3A. In both cases, nasal projections in *Tg{rx3::Gal4}*;UAS:*shh* retinae targeted more anterior regions of the tectum and partially overlapped with projections from the most temporal part of the retina (Fig. 2F in Hernández-Bejarano et al., 2015 and Figure 3A in our manuscript). Even though we cannot discard that some axons reach the posterior region of the tectum, for the sake of comparison with the published data we would like to leave the schematic as it is. We hope the reviewer will agree with our decision.

*Reviewer 2 Advance Summary and Potential Significance to Field:*
*The authors of this paper demonstrate the necessity of Rx3 and FoxD1 in the establishment of the High Acuity Area (HAA) in Zebrafish. It's been shown that knockout of Rx3 leads to a severe disruption of Optic Vesicle (OV) development as well as a loss of FoxD1 expression. Interestingly this reduction was not rescued when the embryos were treated with Shh signaling inhibitor Cyclopamine and FGF signaling inhibitor SU5402. The authors next generated a loss-of-function mutant of FoxD1 to assess its role during the establishment of HAA. They confirmed that this mutant also exhibited naso-temporal axial defects that led to improper axon projection of the Retinal Ganglion Cell (RGC). In addition, the localization of the HAA in these mutants was disrupted. Finally, they associated this disruption with the oculomotor and optokinetic response defects.*

*Reviewer 2 Comments for the Author:*
*While the manuscript is well written and the experiments were well performed and clearly presented, the major concern is the lack of novelty and scientific significance of these results.*

We respectfully disagree with the reviewer's assessment of lack of novelty of this study. We present here complex experiments to determine the genetic interactions controlling *foxd1* expression, and a new loss of function model for *foxd1* in the zebrafish, which allows the analysis of the impact of loss of *foxd1* on visual function. Both approaches are novel and they provide further insight on optic vesicle patterning and *foxd1* function, beyond the studies published up to date. We provide below a more elaborate discussion of these points.

**2a)** *More specifically, the authors demonstrated the necessity of Rx3 for the proper establishment of the FoxG1 and FoxD1 expression boundaries of the OV but did not further investigate the mechanism regarding this phenotype in vivo, as it could be an indirect result due to the severe disruption of OV development (Stigloher 2006).*

A similar concern is presented by Reviewer 1 (comment **1a**), who argues that our results do not allow us to determine whether Rx3 requirement for *foxd1* expression is direct or indirect. As we discuss above, *rx3* is a central factor in the complex gene network controlling eye fate. Removing *rx3* function leads to a deregulation in the expression of many of the other eye specification transcription factors, likely resulting in the eventual loss of eye fate. We thus cannot discard that the loss of *foxd1* expression is due to the loss of eye fate and downstream of the overall misregulation of eye specification transcription factors' expression. Regardless of being direct or indirect, the final effect on *foxd1* expression is clear, and this does have an impact on the establishment of the gene network involving *foxg1*/*foxd1*. We direct the reviewer to the additional data presented in answer **1a)**, and the modified discussion incorporated in the manuscript (page 9; lines 329-335).

**2b)** *While the failure of SU5402 treatment to rescue the expression of FoxD1 is an interesting finding, the requirement of Rx3 for FoxD1 expression has been shown in zebrafish as cited by the authors (Yin 2014).*

The study from Yin et al., 2014 presented a transcriptomic analysis comparing wild type and *rx3* loss of function embryos. *foxd1* is one in a long list of genes shown to be downregulated in *rx3*

© 2022. Published by The Company of Biologists under the terms of the Creative Commons Attribution License (https://creativecommons.org/licenses/by/4.0/).

mutants in this experiment. However, the expression of *foxd1* in *rx3* mutants is not analysed in that study, and the network controlling its expression is not dissected. Thus, our results go beyond the observations presented in that study and provide more detailed information regarding the control of *foxd1* expression during optic vesicle patterning.

**2c)** *Similarly, the characterization of Ephrins in the FoxD1 mutants is elegantly presented, but the role of FoxD1 in the naso-temporal patterning of the retina has also been well studied previously (Carreres 2011).*

Indeed, a mouse model was generated and studied in previous work. However, a model in the zebrafish has never been described before. The mouse and the zebrafish retinae bear some important functional differences. Crucially, the mouse retina does not bear the specialisations associated to visual acuity and colour vision that the zebrafish retina has, so this model allows us to explore further the role of *foxd1* in the establishment of those specialisations – an aspect that cannot be studied in the mouse. Moreover, as mentioned by reviewer 1, the mouse model does not survive postnatally, precluding us from being able to analyse the consequences of Foxd1 absence for visual function. Thus, our model does provide additional insight into *foxd1* function to those provided by mouse model.

**2d)** *Finally, the requirement of FoxD1 for the establishment of HAA, while novel, has not been sufficiently characterized. Importantly, the only evidence for the HAA defect is the subtle reduction in PKC-a expression, which is obscured by uneven exposure and staining as indicated by the DAPI staining. It is important to test other HAA characteristics in these mutants such as the density of the rod photoreceptors as well.*

This concern is shared by all reviewers and we present above (see answers to comments **1c** and **1d**) additional data to support this conclusion. We hope the reviewer will agree with us that new data provide stronger support to our interpretation that the HAA is affected in *foxd1* mutants.

**2e)** *Minor comments:*

*1. The authors claim that FoxG1 expression is expanded throughout most of the prospective eye domain but there is no clear regional delineation in their staining, a co-staining with a pan OV marker such as Six3 could strengthen their argument.*

We believe the results presented in Figure 1 allow to visualise the regional subdivision of the anterior neural plate with a sufficient detail. The phenotypes are fully penetrant, and the comparison of all the images allow to clearly confirm that *foxg1* expansion is within the prospective eye domain. We consider that these additional experiments will not add much to an already very large Figure 1, and would rather maintain the figure as it is.

*2. It would be nice to demonstrate that the DiO-labelled axons of the FoxD1 mutants project throughout the optic track by showing a wholemount picture of these embryos.*

We agree with the reviewer in the importance of determining the impact of the loss of Foxd1 on the optic track to assess the extent of visual pathway disruption in the mutants. As discussed in the answer to comment **1d)**, we now include additional results analysing the optic track, optic chiasm and retinal lamination in the mutants. As described in the answer to comment **1d)**, these results have been incorporated to the manuscript in page 7 and Figure S5.

*3. The number of embryos examined and the penetrance of mutant phenotypes should be indicated in Figures 1 and 2.*

To avoid excessive labelling on the panels in the figures, we had initially opted to present this information in the relevant sections in material and methods. We have now also included these numbers in the figure legends.

*Reviewer 3 Advance Summary and Potential Significance to Field:*
*Hernandez-Bejarano and colleagues have extended their prior work using the zebrafish model system to elucidate further mechanistic insights into the interplay of sonic hedgehog (SHH) and*

© 2022. Published by The Company of Biologists under the terms of the Creative Commons Attribution License (https://creativecommons.org/licenses/by/4.0/).

*fibroblast growth factor (FGF) signalling and the forkhead transcription factor foxd1 towards understanding temporal/nasal patterning and visual system function. Of interest, this interplay requires the rx3 transcription factor. The authors incorporate loss- and gain-of function assays in vivo through the use of contemporary methods to substantiate their work. This work further supports the area for high acuity vision (HAA) in the zebrafish as similar to the fovea in humans and other vertebrates (birds, primates).*

*Reviewer 3 Comments for the Author:*
*Major Concerns:*

**3a)** *Results, Figure 1: The authors should present the data for Cyclopamine and SU5042 separately, as well as combined.*

We agree with the reviewer that the results of individual treatments are relevant. This information was already present in the original manuscript as supplementary information (Figure S2). Even though relevant, we consider the data are not necessary as part of the main figures and we would like to keep them as supplementary information. However, we would consider transforming the figure into a main figure if the reviewers and editor consider it necessary. In that case, we would favour keeping it as an individual figure, since Figure 1 already shows substantial amounts of information.

**3b)** *Results, Figure 2: The use of only one marker, PKCalpha, is not sufficient. At least one additional marker is recommended for assessment of the HAA in the zebrafish.*

This concern is shared by all reviewers. We present above (see answers to comments **1c** and **1d**) additional data addressing this point. We hope the new data provide stronger support to our interpretation that the HAA is indeed affected in *foxd1* mutants.

**3c)** *For the foxd1 mutants, there was very limited characterization of the developing or mature retina. For example, there was no assessment of cell-type specific markers for the 6 neuronal and one glial retinal cell type. This presents a missed opportunity to understand the role of the foxd1 transcription factor in retinal development and function.*

We agree with the reviewer that the *foxd1* mutants provide us with an exciting new model to assess the requirement of *foxd1* for neuronal differentiation. As shown above in answer to comment **1d)**, we have done a preliminary characterisation of retinal markers. Overall, the results suggest that no severe alterations of retinal differentiation are present in the *foxd1* mutants/crispants. We consider that performing the in-depth analysis required to determine more subtle defects goes beyond the scope of this manuscript. We present these results in a new supplementary figure (Figure S5) and include a description of these observations in the manuscript (page 7, line 250-255).

**3d)** *Specific Concerns:*

1. *Results, page 4, lines 122-127: This paragraph should be moved into the Introduction.*

We thank the reviewer for this suggestion, which improves the flow of the manuscript. We have now moved this information to the Introduction (page 3, lines 91-94).

2. *The rx3-/- mutant requires explanation in either the Methods or first use in the Results section.*

Following the reviewer’s recommendation, we have now included a brief description of the *rx3* mutant in results (page 4, lines 123-125).

3. *How were the doses of Cyclopamine and SU5042 determined? Only one dose was used per specific treatment.*

This study is the continuation of a previous study (Hernández-Bejarano et al., 2015), were the same approaches to manipulate Fgf and Shh activity were used. That study describes the

© 2022. Published by The Company of Biologists under the terms of the Creative Commons Attribution License (https://creativecommons.org/licenses/by/4.0/).

optimisation of Shh activity manipulations. Manipulation of Fgf activity was optimised in previous studies from our colleague Alexander Picker (Picker et al., 2005; Picker et al., 2009). Thus, optimisation of these manipulations was not required in this study, since it was already well established. We now include a more explicit mention of the protocols' optimisation in Methods (page 11, lines 414-415).

*4. Although a stop codon is predicted for the foxd1 mutant, the authors haven't established (using assays such as Western blotting) that the protein is actually truncated.*

Indeed, our study does not determine whether a truncated Foxd1 product is generated in the *foxd1* mutant, and we realise now that our statement may lead to confusion. The mutant generated has a 10-basepairs deletion that results in a frameshift and a stop codon in position 70. Our analysis shows a clean, fully penetrant phenotype, consistent with a complete loss of function of the gene. The experiment proposed by the reviewer would not be trivial, since antibodies against the Foxd1 zebrafish protein are not available. We hope the reviewer will agree with our perception that this information is not absolutely necessary for this study, and that the absence of this data does not invalidate the results presented here. To avoid any confusion, we have removed any mention to a putative truncated form of the protein in the relevant part of the text in results (page 5, lines 184-185).

*5. No specific examples of retinotectal projection data was shown; only a summary of pooled data in Figure 3. What was the duration of labelling with DiI and DiO?*

We present the results of DiI/DiO injections in the *foxd1* mutants in Figure 2. The specific experimental details (including the duration of labelling) are described in the methods section (page 11).

*6. The Discussion could be expanded to mention limitations of the OKR and OMR visual function tests and what other tests could be performed using other model systems.*

We already discuss in the manuscript the limitations regarding our morphological and functional analysis of *foxd1* mutants. As mentioned in the manuscript, a *foxd1* mutant exists in the mouse, which shows, similarly to the zebrafish mutant described here, defects in the specification of the temporal region of the retina. However, other aspects of retinal differentiation discussed in our manuscript are not shared, to our knowledge, with the mouse model. Therefore, any other tests performed in mouse will address different aspects of visual function than the ones we analyse in this manuscript.

**3e)** *Other Concerns:*
*1. Abstract: Bring the term "fovea" into the abstract.*

We share with the reviewer the enthusiasm regarding the potential implications of our work for understanding fovea formation. However, even though we think these two structures are functionally comparable, there are also many structural differences. Thus, we hope the reviewer will agree with us that we should be cautious in how far we bring the correlations. Our study focuses on the study of the temporal region of the zebrafish retina and the formation of the HAA in this model organism, and these aspects are appropriately highlighted in the abstract.

---

Second decision letter

MS ID#: DEVELOP/2022/200938

MS TITLE: Foxd1 dependent induction of temporal retinal character is required for visual function

AUTHORS: María Hernández Bejarano, Gaia Gestri, Clinton Monfries, Lisa Tucker, Elena I Dragomir, Isaac H Bianco, Paola Bovolenta, Stephen W Wilson, and Florencia Cavodeassi
ARTICLE TYPE: Research Article

© 2022. Published by The Company of Biologists under the terms of the Creative Commons Attribution License (https://creativecommons.org/licenses/by/4.0/).

I am delighted to tell you that your manuscript has been accepted for publication in Development, pending our standard ethics checks.

<u>Reviewer 2</u>

*Advance summary and potential significance to field*

This manuscript showed that Rx3 and FoxD1 are required for the establishment of the High Acuity Area (HAA) in Zebrafish.

*Comments for the author*

The authors have been attentive to the previous reviews and addressed most questions satisfactorily. It is not clear why they did not measure the cones/rods ratio and density as suggested, which is the most definitive evidence for the HAA area across species. Nevertheless, this revised manuscript provided quantification of UV cone length, which strengthened the argument that Foxd1 regulates the patterning of the HAA. It is sufficient to warrant publication of this study.

<u>Reviewer 3</u>

*Advance summary and potential significance to field*

Reviewer 3 previously summarized the advances made in this paper. In addition to the prior summary, we agree that the foxd1 loss of function model in the zebrafish is a novel contribution.

*Comments for the author*

In the comments to the three peer reviewers and in the revised manuscript, the authors have carefully and comprehensively addressed the concerns raised and performed a limited number of additional experiments and reanalyses where indicated. Hence, the revised manuscript is considerably improved. Moreover, the authors have been pragmatic and in specific instances, partially stepped back from more decisive conclusions not fully supported by the presented data or alternatively, emphasized the strengths and/or limitations of use the murine versus zebrafish foxd1 mutants. Although the authors did not answer this reviewer's specific concern #6, this minor concern does not detract from the manuscript as a whole.

No further revisions to the texts or additional experiments are requested.

© 2022. Published by The Company of Biologists under the terms of the Creative Commons Attribution License (https://creativecommons.org/licenses/by/4.0/).
